# Peripheral GFAP and NfL as early biomarkers for dementia: longitudinal insights from the UK Biobank

**DOI:** 10.1186/s12916-024-03418-8

**Published:** 2024-05-13

**Authors:** Xiaofei Wang, Ziyan Shi, Yuhan Qiu, Dongren Sun, Hongyu Zhou

**Affiliations:** Department of Neurology, West China Hospital, Sichuan University, No.28 Dianxin Nan Street, Chengdu, 610041 China

**Keywords:** Predictive value, Biomarker, Cognitive function, Genetic risk

## Abstract

**Background:**

Peripheral glial fibrillary acidic protein (GFAP) and neurofilament light chain (NfL) are sensitive markers of neuroinflammation and neuronal damage. Previous studies with highly selected participants have shown that peripheral GFAP and NfL levels are elevated in the pre-clinical phase of Alzheimer’s disease (AD) and dementia. However, the predictive value of GFAP and NfL for dementia requires more evidence from population-based cohorts.

**Methods:**

This was a prospective cohort study to evaluate UK Biobank participants enrolled from 2006 to 2010 using plasma GFAP and NfL measurements measured by Olink Target Platform and prospectively followed up for dementia diagnosis. Primary outcome was the risk of clinical diagnosed dementia. Secondary outcomes were cognition. Linear regression was used to assess the associations between peripheral GFAP and NfL with cognition. Cox proportional hazard models with cross-validations were used to estimate associations between elevated GFAP and NfL with risk of dementia. All models were adjusted for covariates.

**Results:**

A subsample of 48,542 participants in the UK Biobank with peripheral GFAP and NfL measurements were evaluated. With an average follow-up of 13.18 ± 2.42 years, 1312 new all-cause dementia cases were identified. Peripheral GFAP and NfL increased up to 15 years before dementia diagnosis was made. After strictly adjusting for confounders, increment in NfL was found to be associated with decreased numeric memory and prolonged reaction time. A greater annualized rate of change in GFAP was significantly associated with faster global cognitive decline. Elevation of GFAP (hazard ratio (HR) ranges from 2.25 to 3.15) and NfL (HR ranges from 1.98 to 4.23) increased the risk for several types of dementia. GFAP and NfL significantly improved the predictive values for dementia using previous models (area under the curve (AUC) ranges from 0.80 to 0.89, C-index ranges from 0.86 to 0.91). The AD genetic risk score and number of APOE*E4 alleles strongly correlated with GFAP and NfL levels.

**Conclusions:**

These results suggest that peripheral GFAP and NfL are potential biomarkers for the early diagnosis of dementia. In addition, anti-inflammatory therapies in the initial stages of dementia may have potential benefits.

**Supplementary Information:**

The online version contains supplementary material available at 10.1186/s12916-024-03418-8.

## Background

With the advent of global aging, Alzheimer’s disease (AD) and dementia impose huge social and economic burden [1, 2]. Currently, the diagnosis of AD and dementia is mainly based on the alteration of specific pathological proteins (such as amyloid and tau proteins) in the central nervous system (CNS) and impaired cognitive functions [3, 4]. However, hidden changes in neuroinflammation and irreversible neuronal damage may occur decades before the onset of symptoms in patients with AD and dementia [5, 6], resulting in delayed diagnostic patterns that contribute to poor clinical prognosis [7, 8]. In addition, there is a lack of effective medications for AD and the lag in the timing of interventions may be an important reason for the poor results of clinical trials [9, 10]. In contrast, interventions during mild cognitive impairment have been proven to be effective in several clinical trials [11], further emphasizing the importance of early diagnosis of AD and dementia.

In 2018, the National Institute on Aging and the Alzheimer’s Association (NIA-AA) introduced a significant paradigm shift in the classification of AD [3]. Departing from traditional reliance on clinical symptomatology, they advocated for a biomarker-based schema, known as the AT(N) system, classifies AD according to the presence of key pathophysiological markers: amyloid-beta (Aβ, A), tau (T), and neurodegeneration (N). This move has prompted research into peripheral biomarkers for early AD and dementia diagnosis, given their practicality and sensitivity compared to cerebrospinal fluid proteins [12–14]. Among them, peripheral glial fibrillary acidic protein (GFAP) and neurofilament light chain (NfL) have gained much traction for their ability to predict the risk of dementia in individuals with subjective cognitive decline and mild cognitive impairment, as well as dementia-specific mortality [15, 16].

GFAP reflects early astrogliosis and neuroinflammation in AD, and recent evidence suggests that it may occur before other well-known pathogeneses [17]. NfL is a classical biomarker of neuronal damage and contributes to the AT(N) biomarker classification scheme [3]. Although both are considered promising biomarkers for the early diagnosis of AD and dementia, their nonspecific expression hinders their clinical application [6, 14, 18]. Elevation of peripheral GFAP and NfL can be observed in other conditions, including traumatic, degenerative, vascular, and autoimmune disorders of the CNS [14, 19]. In addition, factors such as age, sex, and body mass index (BMI) can affect the peripheral expression level [18, 20]. Furthermore, the limited availability of extensive longitudinal cohorts for validation and the specificity of most study criteria further complicate their real-world applicability [21, 22]. Therefore, the diagnostic value of peripheral GFAP and NfL levels should be further explored in dementia-free individuals with larger sample sizes in a population-based cohort.

The UK Biobank, a large and well-characterized real-world cohort, offers a unique vantage point for probing the intricate relationships between GFAP, NfL, and dementia in dementia-free individuals. Utilizing the Olink platform, known for its precision and normalized protein expression (NPX) metric, the UK Biobank provides rigorously standardized data for GFAP and NfL in around 50,000 participants, along with their genetic background and comprehensive medical and socio-environmental data, ensuring its reliability and forming a solid base for in-depth analyses [23].

In our study, we harnessed UK Biobank data to explore the ties between GFAP, NfL, and various dementia types. Over a decade-long follow-up period, we observed elevated GFAP and NfL levels even before dementia diagnosis. Elevated baseline expression was correlated with a higher risk of dementia in dementia-free participants, and the NPX values of GFAP and NfL were linked to a genetic predisposition to dementia.

## Methods

### Study population and sample

**Fig. 1:**
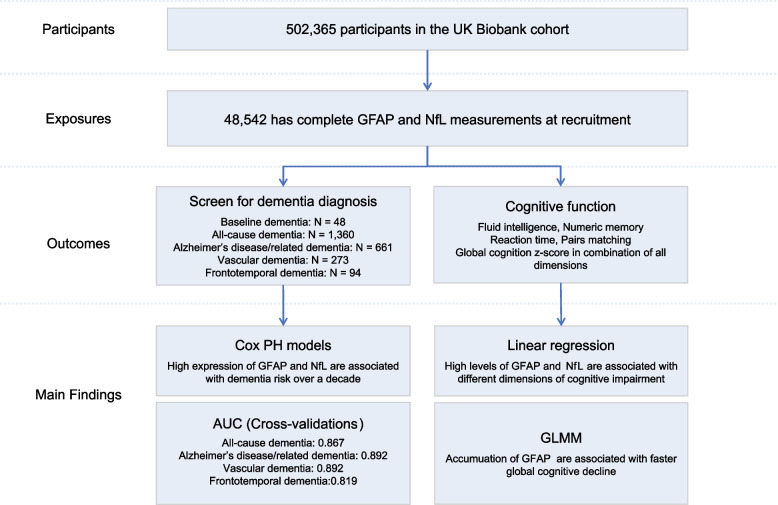
Study design and main findings. (Abbreviations: Cox PH Cox proportional hazard, AUC area under the receiver operating characteristic curves, GLMM generalized linear mixed model)

An overview of the study design is shown in Fig. 1. A prospective UK Biobank cohort study recruited approximately half a million participants across the UK between 2006 and 2010. Plasma protein expression was measured in 52,704 participants using the Olink Target platform; 48,542 of them have complete GFAP and NfL measurements and were enrolled in this study. The sample size for each analysis varied according to the number of participants for whom data were available (Additional file 1: Table S1-S4). Participants were followed up until the date of the first dementia diagnosis, death, loss to follow-up, or date of data censoring (November 2022), with a mean follow-up time of 13.18 ± 2.42 years. The field identifications used in this analysis are described in Additional file 1: Table S1.

### Primary exposures

The expression of plasma GFAP and NfL, contained in the Olink Target Platform panel, were used as the primary exposures. The expression levels of plasma GFAP and NfL were presented as NPX, an Olink’s arbitrary unit in the log2 scale. The advantages of NPX include minimization of both intra- and inter-assay variations, allowing us to identify relative changes in GFAP or NfL levels across participants. The NPX of GFAP and NfL at recruitment and two subsequent visits were extracted for analysis. Details of the Olink data processing can be found in previous publications [24].

### Primary outcomes

The incidence of all-cause dementia was ascertained using diagnoses obtained from the first occurrences of the UKB health outcome datasets (Category 1712, including cases from hospital records, death registration, and primary care) and algorithmically defined outcomes (Category 42). In the first occurrence dataset, the corresponding three-character ICD-10 codes (F00, F01, F02, F03, and G30) were used to define dementia [25]. In the algorithmic datasets, dementia was defined based on the name of the diagnosis. We used the date of diagnosis as the earliest date of the dementia records, irrespective of the source, as previously described [25, 26]. Similar strategies have been used for AD and related dementia (ADRD), vascular dementia (VD), and frontotemporal dementia (FTD). Details of the dementia categorization can be found in Additional file 1: Table S2.

### Cognitive outcomes

Four dimensions of cognition—fluid intelligence, numeric memory (maximum digits remembered correctly), reaction time (mean time to correctly identify matches), and pair matching (average number of incorrect matches) —were selected as reflections of cognitive function. Cognitive outcomes were contemporaneously assessed with the Olink assay during clinic visits (recruitment, instances 2 and 3). Despite the reaction time, the cognitive results were log (measurement + 1) [27–29]. A large fluid intelligence or numeric memory score indicates improved cognitive function [30].

To assess the global view of cognition, we generated weighted z-score combining the above four domains. We first transformed the scores of each cognitive function into z-scores. For cognitive tests like pairs matching and reaction time, where higher values indicate poorer cognitive function, we multiplied the z-scores by − 1 to ensure that higher z-scores consistently indicated better cognitive performance. Since not all participants completed all four cognitive tests (Additional file 1: Table S4), we employed a weighted scoring method to prevent substantial data loss. The weights were assigned based on the number of tests completed by each participant. For instance, if a participant completed all four tests, each test score contributed a weight of 1/4 to the total score. We then summed the weighted z-scores for each participant and re-transformed these sums into a new set of z-scores, as per the method reported previously [31].

### Covariates

To adjust for known confounders that may affect GFAP and NfL expression levels or contribute to dementia, we employed three models in the following analysis. Model 1 was adjusted for age, age squared (to accommodate a potential curvilinear relationship between age and dementia diagnosis and cognition decline), sex, and body mass index (BMI). Model 2 encompassed all covariates from Model 1 and further integrated socio-environmental confounders, including smoking status (categorized as never, former, or current smoker), alcohol consumption frequency (scored from 0 to 5 based on intake regularity), Townsend deprivation index (TDI) at recruitment, educational attainment (bifurcated as high school equivalent or UK A levels) [26], frequency of moderate physical activity (at least 10 min/week), self-reported racial background (Asian, Black, White, or multiracial, serving as a surrogate for variations in life course experiences), and the count of apolipoprotein E (APOE)*E4 alleles present. Model 3 was further adjusted for prevalent comorbidities, including diabetes, hypertension, cerebrovascular conditions, demyelination, neurodegenerative disorders, organic brain diseases, and mental health disorders. Detailed categorization and information on missing values can be found in Additional file 1: Table S2–S3.

### Statistical analysis

Statistical analyses were conducted using R software (version 4.2.2, R Project for Statistical Computing). A two-tailed *P*-value of < 0.05 was deemed statistically significant. Using the Cox proportional hazard (PH) models from the *survival* package (version 3.5–5), we ascertained the hazard ratio of dementia in relation to elevated GFAP and NfL NPX, adjusting for the aforementioned confounders. GFAP and NfL were categorized into binary variables in Cox PH models according to their quartile of expression levels. We examined the associations of baseline GFAP and NfL levels (as independent variables) with cognitive measures using linear regression models, controlling for confounders. Similarly, linear regression models explored the link between the genetic risk score for AD (AD-GRS) or the number of APOE*E4 alleles (as independent variables) and protein expressions (as dependent variables), controlling for age, age squared, sex, BMI, race, and the first ten genetic principal components related to genetic population stratification [26, 32].

To explore the predictive values of GFAP and NfL for dementia in dementia-free participants [33], Cox PH models with internal leave-one-region-out cross-validation were employed [34]. The cohort was divided based on the 22 assessment centers of UK Biobank, and we selected participants from one center as the test set and those from the remained centers for model training. This process was iteratively repeated for each center. Due to the limited number of FTD cases, the 22 centers were grouped into threefolds for cross-validation. The corresponding areas under the receiver operating characteristic curves (AUC) were generated using the *pROC* package (version 1.18.2). C-Index was derived from the *survival* package of the training models. Two established predictive models for dementia: the Cardiovascular Risk Factors, Aging, and Incidence of Dementia Risk Score (CAIDE) [35] and the Dementia Risk Score (DRS) [36] were compared for the efficacy in dementia prediction, both individually and combined with GFAP and NfL levels. CAIDE model utilizes age (grouped by 47), sex, BMI (grouped by 30), education level, hypertension, hypercholesterolemia (defined by total cholesterol ≤ 6.5 mmol/L), and APOE*E4 allele number. DRS model utilizes age, sex, BMI, social deprivation, smoking status, alcohol intake status, current usage of anti-hypertensive drugs, usage of aspirin, diabetes, cerebrovascular conditions, atrial fibrillation, and depressive disorders. We modified the DRS model by adding APOE*E4 allele numbers (DRSm). Net reclassification index (NRI) with risk thresholds of 20% and 60% were computed using *nricens* package (version 1.6), applying 100 bootstrap iterations without cross-validation [37].

To elucidate the trajectory of GFAP and NfL levels preceding dementia diagnosis, a backward time scale was adopted, setting time 0 as either the point of dementia diagnosis or the end of follow-up. Data visualization was performed using *ggplot2* package (version 3.4.2), applying loess regression with a 95% confidence interval [38]. Sensitivity analyses included subgroup analysis by age, sex, BMI, self-reported racial background, genetic background (AD-GRS, number of APOE*E4 alleles), and comorbidities for incident all-cause dementia by using Cox PH model. We also exclude participants with prevalent comorbidities as described earlier and using Cox PH models to confirm the hazard ratios of dementia in relation to elevated GFAP and NfL levels. Missing covariate data were imputed using the mode for categorical variables and multiple imputation by *mice* package (version 3.15.0) for continuous variables; subsequently, the Cox PH model for all-cause dementia was reanalyzed to assess the robustness of our findings.

## Results

### Demographics

The study design and primary outcomes are shown in Fig. 1. A total of 48,542 participants from the UK Biobank cohort has complete GFAP and NfL measurements assessed using Olink’s assay from blood samples obtained at recruitment (mean age: 56.8 ± 8.21 years) (average follow-up duration: 13.18 ± 2.42 years). Demographic details of the participants are presented in Table 1 and Additional file 1: Table S4. We identified 1360 all-cause dementia cases, of which 1312 were diagnosed after recruitment. Participants with dementia diagnosis before recruitment are relative younger than those diagnosed after recruitment (Additional file 1: Fig. S1). The mean duration to dementia diagnosis was 8.35 ± 4.09 years, with the longest duration being 15.21 years. The average age of a dementia diagnosis made after recruitment was 73.39 ± 6.16 years.

**Table 1:** Baseline demographic characteristics of participants enrolled in UK Biobank with GFAP and NfL expressions

	Total (*N* = 48,542)
Sex (female), *n* (%)	26,152 (53.9)
Age in yeas, mean (SD)	56.8 (8.21)
BMI in kg/m^2^, mean (SD)	27.5 (4.79)
GFAP NPX, median (min, max)	0 [− 2.24, 6.43]
NfL NPX, median (min, max)	0 [− 2.98, 5.15]
Townsend deprivation index, median [min, max]	− 2.05 [− 6.26, 10.4]
High school equivalent or more schooling, *n* (%)	31,231 (64.3)
Days per week of moderate physical activity, mean (SD)	3.62 (2.34)
Former or current smoker, *n* (%)	22, 239 (45.8)
Alcohol frequency, *n* (%)	
Never, *n* (%)	4198 (8.6)
Special occasions only, *n* (%)	5692 (11.7)
One to three times a month, *n* (%)	5256 (10.8)
Once or twice a week, *n* (%)	12,596 (25.9)
Three or four times a week, *n* (%)	10,901 (22.5)
Daily or almost daily, *n* (%)	9782 (20.2)
Race	
White, *n* (%)	45,269 (93.3)
Black, *n* (%)	1252 (2.6)
Asian, *n* (%)	1104 (2.3)
Multiracial, *n* (%)	681 (1.4)
APOE*E4 carrier, *n* (%)	
E2E4 & E3E4	10,682 (22.0)
E4E4	1193 (2.5)
Comorbidity, *n* (%)	
Diabetes, *n* (%)	2743 (5.7)
Hypertension, *n* (%)	13,651 (28.1)
Cerebrovascular disorders, *n* (%)	1223 (2.5)
Demyelinating disorders, *n* (%)	382 (0.8)
Neurodegenerative disorders, *n* (%)	473 (1.0)
Organic brain diseases, *n* (%)	484 (1.0)
Mental disorders, *n* (%)	7746 (16.0)
Cognition	
Fluid intelligence, log (measurement + 1), mean (SD)	1.88 (0.35)
Numeric memory, log (measurement + 1), mean (SD)	2.02 (0.19)
Pairs matching, log (measurement + 1), mean (SD)	1.08 (0.53)
Reaction time in ms, mean (SD)	564 (123)
Dementia diagnosis in total	
All-cause dementia, *n* (%)	1360 (2.8)
Alzheimer’s disease and related dementia, *n* (%)	661 (1.4)
Vascular dementia, *n* (%)	273 (0.6)
Frontotemporal dementia, *n* (%)	94 (0.2)

Missing values are reported in supplementary data

*BMI* Body mass index, *GFAP* Glial fibrillary acidic protein, *NfL* Neurofilament light chain, *NPX* Normalized protein expression, *APOE* Apolipoprotein, *ms* Milliseconds

The distribution of peripheral GFAP and NfL within our study cohort is detailed in Additional file 1: Fig. S2 and Additional file 1: Table S5-S6. Aligning with previous findings, we observed that age, BMI, and racial background significantly influenced GFAP and NfL expression levels, and these levels varied in the presence of certain medical conditions including diabetes, hypertension, cerebrovascular disorders, demyelination, neurodegeneration, organic brain diseases, and mental health disorders. Intriguingly, our analysis revealed that individuals exhibiting higher levels of GFAP and NfL (falling within the highest quartile) were more likely to be carriers of the APOE*E4 allele.

In consideration of the observation that healthier individuals are more likely to complete repeated measurements, we categorized participants based on their follow-up protein measurement data (Additional file 1: Table S7). Notably, a vast majority, 97.68% (47,418 out of 48,542), had only baseline measurements of GFAP and NfL. Participants with subsequent protein measurements tended to be younger (mean age 50.2 years compared to 57.0 years, *P* < 0.001), more educated (82.5% versus 63.9%, *P* < 0.001), and exhibited healthier lifestyles (as indicated by smoking history, 37.9% versus 46.0%, *P* < 0.001), alongside fewer comorbid conditions. More importantly, these individuals not only demonstrated enhanced cognitive functioning and lower GFAP and NfL levels but also remained free of any dementia diagnosis throughout the duration of the study.

### Higher levels of peripheral GFAP and NfL associated with cognition and dementia risk

**Fig. 2:**
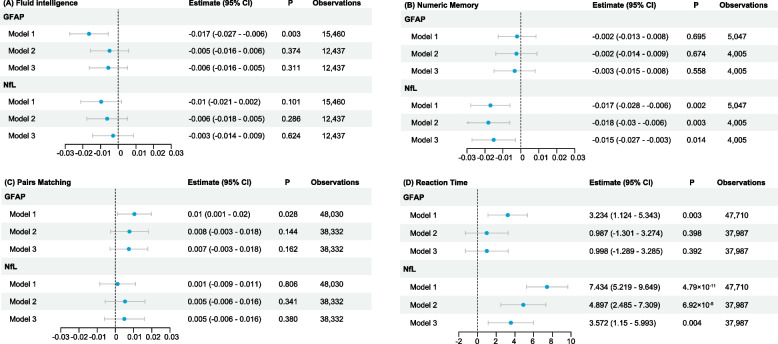
Linear regression estimates for the association between peripheral GFAP, NfL, and cognitive function

Figure 2 delineates the relationship between peripheral GFAP and NfL levels and cognitive measurements, with demographic characteristics presented in Additional file 1: Table S4. The influence of GFAP and NfL on various cognitive dimensions is evident. Elevated GFAP levels were associated with poor fluid intelligence (model 1, estimate =  − 0.017, 95% CI =  − 0.027 to − 0.006), prolonged reaction times (model 1, estimate = 3.23, 95% CI = 1.12 to 5.34), and suboptimal pair-matching results (model 1, estimate = 0.01, 95% CI = 0.001 to 0.020). Elevated NfL levels significantly correlate with decreased numeric memory (model 3, estimate =  − 0.015, 95% CI =  − 0.028 to − 0.006) and prolonged reaction time (model 3, estimate = 3.57, 95% CI = 1.15 to 5.99). Using a global z-score for combining different domains of cognition, significant negative associations were found for both GFAP (model 3, estimate =  − 0.021, 95% CI =  − 0.04 to − 0.002) and NfL (model 3, estimate =  − 0.026, 95% CI =  − 0.045 to − 0.006) (Additional file 1: Fig. S3-S5).

**Fig. 3:**
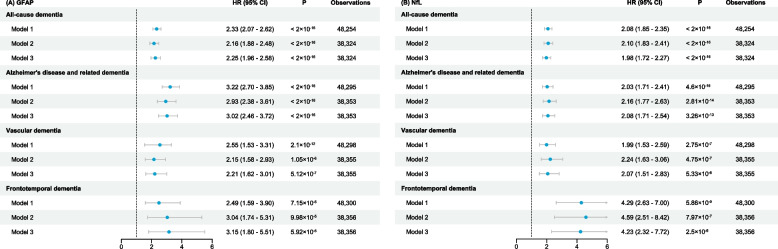
Hazard ratios for dementia associated with peripheral GFAP and NfL

To find the cutoff values for GFAP and NfL associated with an elevated risk of dementia, we grouped their expression by quartiles and found that interval 4 of both GFAP and NfL significantly increased the risk of developing dementia (Additional file 1: Table S8), and the cutoff values at this point were 0.363 for GFAP and 0.353 for NfL. Participants with GFAP levels exceeding 0.363 faced a significantly increased hazard of developing all-cause dementia (model 3, hazard ratio (HR) = 2.25; 95% CI = 1.96 to 2.58) (Fig. 3A). High GFAP levels were also associated with an increased risk of developing other types of dementia (HR = 3.02, 2.21, and 3.05 for ADRD, VD, and FTD, respectively) (Fig. 3A). Similarly, significant associations are noted for high expression of NfL (> 0.353) with the hazard of developing all cause-dementia, ADRD, and VD (HR = 1.98, 2.08, and 2.07, respectively) (Fig. 3B). High expression of NfL showed more risk in developing FTD (model 3, HR = 4.23; 95% CI = 2.32 to 7.72) (Fig. 3B). Taken together, higher peripheral GFAP and NfL levels are independent risk factors for cognitive decline and dementia in dementia-free participants.

### Early elevation of peripheral GFAP and NfL levels shared genetic factors with AD

The participants were divided into three groups based on their dementia diagnosis: never (no dementia record in the cohort, *N* = 47,182), potential (dementia diagnosis made after recruitment, *N* = 1,360), and existing (already diagnosed with dementia at recruitment, *N* = 48). We noted significant elevations in GFAP and NfL NPX levels when comparing participants in the never group to those in the existing group; importantly, participants in the potential group demonstrated GFAP and NfL NPX levels similar to those in the existing group (Additional file 1: Fig. S6). After matching for age and sex using the propensity score matching method at a ratio of 1:10, significant differences remained (Additional file 1: Fig. S7). In accordance with the results shown in Fig. 2, cognitive decline was observed in the potential group (Additional file 1: Fig. S8).

**Fig. 4:**
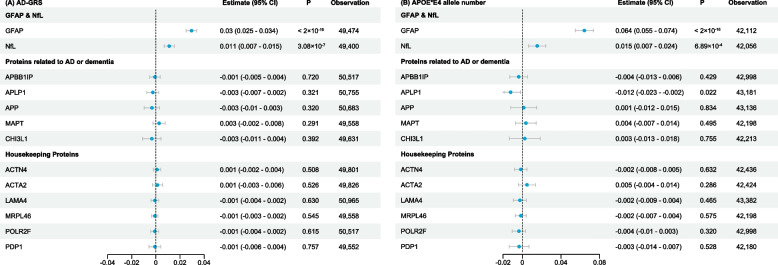
Correlation between peripheral proteins and genetic tools for Alzheimer’s disease

Previous studies have suggested that the increase in GFAP in the pre-clinical phase of AD is independent of classical AD pathology [39, 40]. We therefore used two genetic tools for AD, the AD-GRS and the number of APOE*E4 alleles carrying as independent variables, to explore the association between genetic of dementia and GFAP or NfL. We found a significant association between the AD-GRS and GFAP (estimate = 0.03, 95% CI = 0.025 to 0.034) and NfL (estimate = 0.011, 95% CI = 0.007 to 0.015) (Fig. 4A). We also found a significant association between the number of APOE*E4 alleles and GFAP (estimate = 0.064, 95% CI = 0.055 to 0.074) and NfL (estimate = 0.015, 95% CI = 0.007 to 0.024) (Fig. 4B). For proteins implicated in the pathogenesis of AD and dementia, including amyloid beta precursor protein binding family B member 1 (APBB1IP), amyloid beta precursor like protein 1 (APLP1), amyloid beta precursor protein (APP), and microtubule associated protein tau (MAPT), only trends without statistical significance were observed in relation to AD-GRS. Similar results were found when using APOE*E4 alleles as genetic tools, except for APLP1 which showed a negative association. Chitinase 3 Like 1 (CHI3L1), previously reported to be a differentially expressed protein in AD and dementia, also showed no association with the genetic tools for AD. In addition, no associations were observed between AD-GRS or APOE*E4 alleles and several housekeeping proteins used as controls since they are often stably expressed in different physiological or pathological conditions. Taken together, these results suggest that early neuroinflammation and neuronal damage, reflected by elevated peripheral GFAP and NfL expression, may be genetically determined in patients at high risk of AD.

### Association between the longitudinal changes of peripheral GFAP and NfL with dementia progression

For participants who later manifested dementia, the time to diagnosis was significantly associated with GFAP and NfL levels, but this may be a false positive result due to the large sample size (Additional file 1: Fig. S9). We then used a backward time scale to observe the trajectory of GFAP and NfL levels prior to the onset of dementia (Additional file 1: Fig. S10-S11). In line with previous findings, peripheral GFAP and NfL are increased with advancing age. Pearson correlation analyses also reveal positive correlation between GFAP (*r* = 0.377, *P* < 0.001) and NfL expression (*r* = 0.501, *P* < 0.001) with age (Additional file 1: Table S9). By setting the time of dementia diagnosis (for those with dementia) or time of end of follow-up (for those without dementia) as 0-time mark, we discerned notable disparities in the expression levels of GFAP and NfL up to 15 years before diagnosis.

It should be noted that the 97.7% (47,418 of 48,542) of participants in this study had a single protein measurement (Additional file 1: Table S7). The remain 1124 participants with repeated protein measurements were healthier in respect of the risk factors for dementia, including younger age, smaller BMI, higher education level, fewer smoking status, and less comorbidities, and they presented superior cognitive performances. This subset provided a unique opportunity to examine the relationship between the accumulation rates of peripheral GFAP and NfL and the rate of cognitive decline preceding dementia and early cognitive impairment. We found accelerated annual change in GFAP was significantly associated with more rapid cognitive decline (model 3, estimate =  − 0.101, 95% CI = 0.163 to − 0.040), and the interaction between GFAP expression and follow-up time was not statistically significant. However, we did not observe associations between accumulation of NfL with global cognitive decline (Additional file 1: Table S10-S11).

These results suggest that peripheral GFAP and NfL levels, as well as the accumulation rate of GFAP, are associated with the progression of dementia, further emphasizing the potential of early interventions targeting neuroinflammation in dementia.

### High predictive value by using baseline GFAP and NfL NPX for dementia

**Fig. 5:**
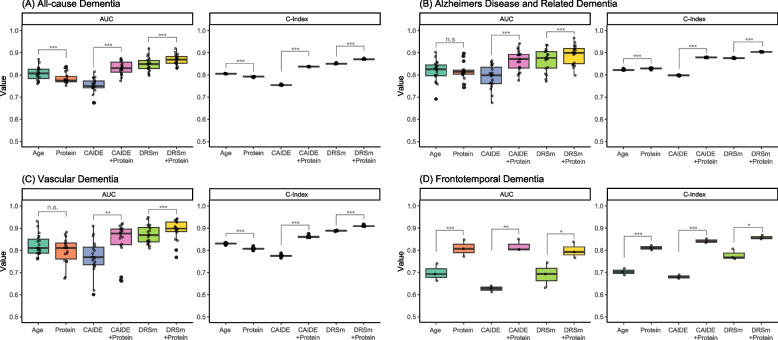
Predictive value of peripheral GFAP and NfL for dementia. n.s. not significant, *P < 0.05, **P < 0.01, ***P < 0.001 by paired *t*-test)

We evaluated the predictive value of baseline GFAP and NfL levels for dementia in dementia-free participants with leave-one-region out validation, as shown in Fig. 5 and Additional file 1: Table S12. Using GFAP and NfL alone achieved AUC in predicting dementia of 0.781 to 0.816, with corresponding C-Index of 0.792 to 0.829. Compared to using age alone as the predictor for dementia, incorporating GFAP and NfL improved the NRI for predicting all-cause dementia, ADRD, and VD (NRI = 0.012 to 0.088). Furthermore, the predictive values were higher when using GFAP and NfL alone compared to CAIDE model. When adding GFAP and NfL to CAIDE and DRSm models, the predictive value significantly improved in predicting all-cause dementia and ADRD (NRI = 0.128 to 0.173). The best model for predicting all-cause dementia, ADRD, and VD was DRSm combined with GFAP and NfL, with corresponding AUC of 0.867, 0.892, and 0.892 respectively, and corresponding C-Index of 0.871, 0.904, and 0.910. The combination of GFAP and NfL with established models did not improve the efficacy in predicting FTD (NRI =  − 0.0002).

### Sensitivity analysis

**Fig. 6:**
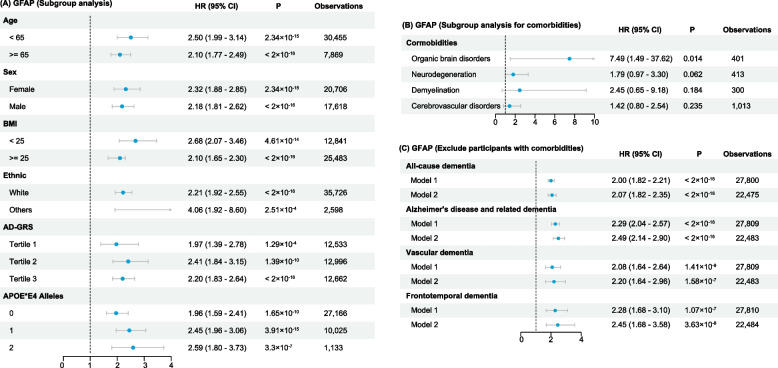
Subgroup analysis of the hazard ratios for all-cause dementia associated with GFAP

**Fig. 7:**
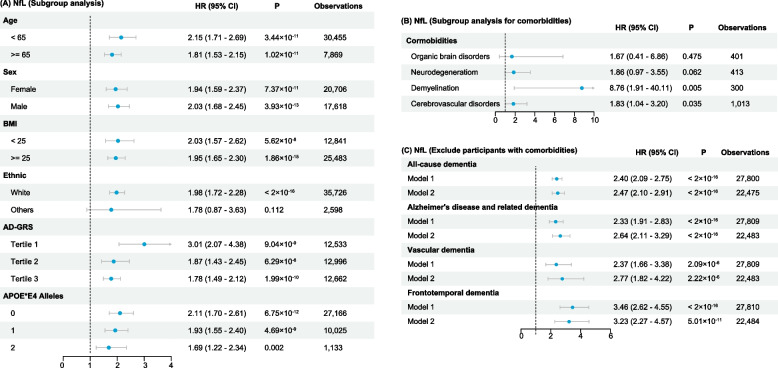
Subgroup analysis of the hazard ratios for all-cause dementia associated with NfL

We performed subgroup analyses for age, sex, BMI, self-reported racial background, AD-GRS, and number of APOE*E4 alleles for incident all-cause dementia (Figs. 6A and 7A), and we found the results were consistent with our primary findings (Fig. 3) (except for NfL that did not show significance in non-White participants), indicating the increment of both GFAP and NfL across different subgroups suggests the risk of incident dementia. Interestingly, upon stratification by AD-GRS and number of APOE*E4 alleles, we noticed that GFAP demonstrated stronger correlation in the high-risk participants, while NfL showed stronger correlation in the low-risk participants.

When examining subgroups with distinct comorbidities, we observed that elevated GFAP levels heightened the risk of dementia among individuals with organic brain disorders. Although similar trends were seen in those with neurodegenerative disorders, demyelination, and cerebrovascular disorders, the associations did not reach statistical significance (Fig. 6B). Conversely, increased NfL levels were significantly associated with a higher risk of dementia in cases of demyelination and cerebrovascular disorders (Fig. 7B). These patterns may be attributed to the non-specific nature of GFAP and NfL, which broadly indicate neuroinflammatory and neurodegenerative changes. Nonetheless, they appear to mirror the shared pathological shifts accompanying dementia comorbid with certain neurological conditions. We then strictly included dementia-free participants who did not have comorbidities that were mentioned in our model 3 (Figs. 6C and 7C), and we found the results of these analyses were consistent with our primary findings.

In conducting a competing risk analysis, where non-dementia deaths were treated as competing events, we observed a slightly reduced HR for dementia in relation to GFAP, while an increase in HR was noted for NfL (Additional file 1: Table S13). Acknowledging that missing data related to BMI, lifestyle factors, and genetic risks could potentially skew our results, we carried out imputations for these variables and re-analyzed our dataset. The re-evaluation confirmed the robustness of the dementia risks associated with GFAP and NfL (Additional file 1: Table S14).

Furthermore, we refined our mixed model by integrating follow-up durations as random slopes, which reinforced the finding that higher rates of GFAP accumulation were significantly linked to accelerated cognitive decline (Additional file 1: Table S10).

Finally, we stratified our participants into two age groups (< 65 and ≥ 65 years) and evaluated the predictive efficacy of GFAP and NfL (Additional file 1: Table S15-S16). Overall, we found that GFAP and NfL provided higher predictive values for dementia in participants < 65 years old compared to those ≥ 65 years old. Using GFAP and NfL as the predictors was more effective in predicting all-cause dementia and ADRD compared to using age alone. Furthermore,the predictive efficacy of the DRSm model augmented with protein measures revealed enhanced predictive accuracy in participants under 65 years of age (Additional file 1: Table S16).

## Discussion

The latest AT(N) biomarker classification framework underscores the significance of peripheral biomarkers in diagnosing and monitoring disease progression in AD [3, 4]. Among these biomarkers, GFAP and NfL are considered non-specific but important biomarkers of AD pathogenesis. GFAP is a major cytoskeletal component of astrocytes. Reactive astrocytosis, represented by elevated peripheral GFAP, has been recognized as a potential driver of AD. NfL is one of the most abundant components of myelinated axons and is released into the periphery under neuronal damage [14, 22]. Although both are associated with a higher risk of incident dementia and faster rates of cognitive decline [41, 42], their non-specificity for AD and dementia limits further clinical application [19, 43, 44]. Several factors such as age, sex, BMI, and race affect the expression of GFAP and NfL [18, 20]. Elevated peripheral GFAP and NfL levels can also be observed in other conditions, including traumatic brain disorders, neurodegenerative disorders (e.g., Parkinson disease and amyotrophic lateral sclerosis), and autoimmune disorders of the CNS (e.g., multiple sclerosis) [14, 19]. In addition, most studies on the predictive values of peripheral GFAP and NfL were based on selected participants, leaving their roles in the general population poorly understood [21, 22].

To overcome these shortcomings, we used a well-documented UK Biobank cohort and evaluated the diagnostic value of peripheral GFAP and NfL levels in a dementia-free population after strict adjustment for multiple confounders. Consistent with previous studies, we found that elevated peripheral GFAP and NfL levels are associated with cognitive impairment and incident dementia and are notably effective in distinguishing participants with dementia. More importantly, using the AD-GRS and APOE*E4 allele number, we identified potential shared genetic factors between dementia, GFAP, and NfL expression.

In our study, peripheral GFAP and NfL levels were notably effective in distinguishing participants with dementia from others, corroborating the findings of previous studies. Shen et al. [39]observed that peripheral GFAP levels could be instrumental in the early diagnosis of AD. Gao et al. [45] associated peripheral GFAP levels with cognitive decline and brain atrophy. Rajan et al. [46]noted the rapid accumulation of peripheral GFAP in individuals developing clinical AD. Sarto et al. [47] highlighted the diagnostic accuracy of peripheral GFAP levels in differentiating AD from non-neurodegenerative cases. Increased peripheral concentrations of NfL have been linked to hastened progression to AD dementia onset [21] and have demonstrated high accuracy in differentiating FTD from healthy participants [47]. Nevertheless, as of now, there has been no research validating the relationship between GFAP and NfL with incident dementia in a large-scale population. We discovered that when combining GFAP and NfL with published models, the predicting values are obviously improved in for all-cause dementia and ADRD. While there is a marginal improvement in predicting VD, these two proteins did not demonstrate a notable enhancement in the prediction of FTD. This suggests a need for further research to identify specific risk factors for FTD. Overall, our findings underscore their potential as sensitive and cost-effective biomarkers for dementia.

While abundant evidence underscores the role of neuroinflammation in the pathogenesis of AD, its position as a cause and consequences remain undetermined [48]. Besides the early changes of inflammatory proteins, GFAP has also been linked to brain Aβ pathology [49]. Recently, reports suggest that astrocyte reactivity, marked by elevated peripheral GFAP, is an upstream event linking Aβ with initial tau phosphorylation in pre-clinical AD [40]. Evidence from a large genome-wide association study has also shown a causal relationship between neuroinflammation and ADRD [50]. However, neuroinflammation can be triggered by the neurodegenerative processes of AD, such as tau dysfunction and Aβ deposition [48, 51, 52], potentially rendering it a non-specific response. In our study with ~ 50,000 participants, we found robust correlations between peripheral GFAP and NfL expression with AD-GRS APOE*E4 alleles, suggesting shared genetic factors driving early neuroinflammation and neurodegenerative changes in dementia. Similar findings have been previously reported [53–56]. Furthermore, our analysis did not identify any significant associations between several peripheral proteins linked to the pathogenesis of AD and dementia. Based on the aforementioned findings, it could be inferred that neuroinflammation, along with neuronal damage within the brain, precedes dementia and is influenced by underlying genetic factors.

Elevated peripheral GFAP and NfL levels can be observed during the early or asymptomatic phase of AD [13]. Our findings corroborate that an increase in peripheral GFAP and NfL levels can manifest over a decade prior to the diagnosis of dementia [13]. Notably, alterations in cognitive function were observed in this subset of participants. When evaluating longitudinal changes, significant correlations were observed between the faster-annualized rate of change in GFAP and cognition, suggesting that the accumulation of peripheral GFAP reflects the severity of dementia [39, 57]. It should be noted that the missing protein measurements during follow-up may limit our findings. Nevertheless, together with the strong association between GFAP, NfL, and AD-GRS, our results provide a basis for applying anti-inflammatory and neuroprotective therapies as early interventions for AD in clinical practice [13].

This study had several strengths. The UK Biobank is a large cohort of middle-aged adults with data on GFAP and NfL expression levels, complete cognitive function, and related medical records. The large sample size bolsters the evidence supporting peripheral GFAP and NfL as early biomarkers of dementia. To the best of our knowledge, this is the first and largest study to simultaneously evaluate the association between GFAP and NfL and the risk of dementia and cognition in a population-based cohort.

However, several limitations should be noted when interpreting the results. A significant limitation arises from the inherent characteristics of the UK Biobank cohort. The participants enrolled in the UK Biobank tend to represent a more health-conscious and educated segment of the population. This selection bias may influence the findings, as the sample might not fully represent the general population’s diversity, particularly in terms of overall health status and educational background. Such a bias could potentially affect the generalizability of our results to broader, more varied populations. Second, the average age of participants at the time of enrollment was around 50 years, with a follow-up duration of 13 years. This relatively younger cohort and shorter follow-up period may not be entirely representative of the typical age range at which dementia is most prevalent, which is usually around 80 to 90 years [2, 58]. Consequently, our study might have captured only a limited spectrum of dementia cases, potentially missing out on the majority of late-onset cases that occur in older age groups. This factor could limit the comprehensiveness of our findings in the context of dementia epidemiology. Third, only a limited subset of participants underwent GFAP and NfL NPX measurements at various time points, which hindered the ability to evaluate changes within an individual in relation to GFAP and NfL levels. In addition, although we adjusted for multiple confounders, the missing values for some variables may have caused false positives in our models. In our study, we used genetic instruments specific to AD. This approach was based on the premise that many genetic pathways and risk factors for AD may also be relevant to other forms of dementia, given the overlapping pathophysiological features among various dementia types [26, 32, 59]. However, this assumption warrants a cautious interpretation, especially when generalizing findings to all forms of dementia. Finally, the diagnosis of dementia is registry-based, limiting the exploration of the relationship between GFAP, NfL, and other compulsory factors for dementia diagnosis.

## Conclusions

In summary, our data demonstrated that elevated levels of peripheral GFAP and NfL are associated with cognitive impairment and incident dementia. Our findings underscore the potential utility of peripheral GFAP and NfL levels in the early diagnosis of dementia and suggest a potential role for anti-inflammatory therapies in the early phase of dementia.

### Supplementary Information

Additional file 1: Figures S1-S11. Fig S1. Distribution of Age at Dementia Diagnosis. Fig S2. Distribution of GFAP and NfL Expression at Different Visits. Fig S3. Distribution of Cognitive Measurements at Different Visits. Fig S4. Distribution of Global Cognition Z-Score at Different Visits. Fig S5. Linear Regression Analysis For GFAP and NfL Expression with Global Cognition. Fig S6. Comparison of Peripheral GFAP and NfL Expression Level According to the Diagnosis of Dementia. Fig S7. Comparison of Peripheral GFAP and NfL Expression Level According to the Diagnosis of Dementia after Propensity Score Matching. Fig S8. Comparison of Baseline Cognitive Measurements Between Never and Potential Dementia Groups after Propensity Score Matching. Fig S9. Correlation Between Time to a Dementia Diagnosis with Baseline Peripheral GFAP and NfL. Fig S10. Trajectories of GFAP Expression Over 15 Years Preceding Diagnosis of Dementia Using Loess Regression. Fig S11. Trajectories of NfL Expression Over 15 Years Preceding Diagnosis of Dementia Using Loess Regression. Tables S1-S16. Table S1. Field IDs Used in Analysis. Table S2. Data Categorization. Table S3. Missingness at Baseline for the Cohort. Table S4. Baseline Demographic Characteristics Among Individuals Enrolled in UK Biobank and Evaluated for Olink’s Assay and Cognition. Table S5. Baseline Characteristics Grouped by GFAP Quartile. Table S6. Baseline Characteristics Grouped by NfL Quartile. Table S7. Comparison of Baseline Characteristics Between Participants with Multiple Protein Measurements and Single Protein Measurement. Table S8. Hazard Ratios for All-cause Dementia According to GFAP and NfL Quartiles. Table S9. Correlation Between GFAP and NfL with Age. Table S10. Association Between Annualized Change Rate of GFAP and NfL with Annualized Change Rate of Global Cognition. Table S11. Association Between Annualized Change Rate of GFAP and NfL with Annualized Change Rate of Global Cognition (Setting Follow-up Time as Random Slope). Table S12. Values of Predictive Models Under Leave-One-Region-Out Validations. Table S13. Competing Risk Analysis. Table S14. Sensitivity Analysis for the Association Between GFAP and NfL With Incident All-cause Dementia (Imputing Missing Values). Table S15. Predictive Model Using Age or Protein Expressions and Stratified by Age Groups. Table S16. Predictive Model Using DRSm Combined with Protein Expression and Stratified by Age Groups.

## Data Availability

The usage of UK Biobank data has been approved by UK Biobank Research Team (Application ID:98826). Raw data of this study is available after approval by the UK Biobank Research Team. For the purpose of verification, validation, replication, or meta-analyses, the external researcher can contact the corresponding author.

## Abbreviations

AD: Alzheimer’s disease
ADRD: AD and related dementias
AD-GRS: Genetic risk score for AD
APOE: Apolipoprotein E
AUC: Areas under the curve
FTD: Frontotemporal dementia
GFAP: Glial fibrillary acidic protein
NfL: Neurofilament light chain
NPX: Normalized protein expression
NRI: Net reclassification index
ROC: Receiver operating characteristic curves
VD: Vascular dementia
