# Supplementary material for: Peripheral GFAP and NfL as early biomarkers for dementia: longitudinal insights from the UK Biobank
Source: BMC Med. 2024 May 13;22:192. doi: 10.1186/s12916-024-03418-8 (PMC11089788; doi:10.1186/s12916-024-03418-8)
Supplement: Supplementary file 1 — Additional file 1: Figures S1-S11. Fig S1. Distribution of Age at Dementia Diagnosis. Fig S2. Distribution of GFAP and NfL Expression at Different Visits. Fig S3. Distribution of Cognitive Measurements at Different Visits. Fig S4. Distribution of Global Cognition Z-Score at Different Visits. Fig S5. Linear Regression Analysis For GFAP and NfL Expression with Global Cognition. Fig S6. Comparison of Peripheral GFAP and NfL Expression Level According to the Diagnosis of Dementia. Fig S7. Comparison of Peripheral GFAP and NfL Expression Level According to the Diagnosis of Dementia after Propensity Score Matching. Fig S8. Comparison of Baseline Cognitive Measurements Between Never and Potential Dementia Groups after Propensity Score Matching. Fig S9. Correlation Between Time to a Dementia Diagnosis with Baseline Peripheral GFAP and NfL. Fig S10. Trajectories of GFAP Expression Over 15 Years Preceding Diagnosis of Dementia Using Loess Regression. Fig S11. Trajectories of NfL Expression Over 15 Years Preceding Diagnosis of Dementia Using Loess Regression. Tables S1-S16. Table S1. Field IDs Used in Analysis. Table S2. Data Categorization. Table S3. Missingness at Baseline for the Cohort. Table S4. Baseline Demographic Characteristics Among Individuals Enrolled in UK Biobank and Evaluated for Olink’s Assay and Cognition. Table S5. Baseline Characteristics Grouped by GFAP Quartile. Table S6. Baseline Characteristics Grouped by NfL Quartile. Table S7. Comparison of Baseline Characteristics Between Participants with Multiple Protein Measurements and Single Protein Measurement. Table S8. Hazard Ratios for All-cause Dementia According to GFAP and NfL Quartiles. Table S9. Correlation Between GFAP and NfL with Age. Table S10. Association Between Annualized Change Rate of GFAP and NfL with Annualized Change Rate of Global Cognition. Table S11. Association Between Annualized Change Rate of GFAP and NfL with Annualized Change Rate of Global Cognition (Setting Follow-up Time as Random [file 12916_2024_3418_MOESM1_ESM.docx]

# Additional File 1

## Supplementary Methods

## Figures S1-S11

**Figure S1.** Distribution of Age at Dementia Diagnosis.

**Figure S2.** Distribution of GFAP and NfL Expression at Different Visits.

**Figure S3.** Distribution of Cognitive Measurements at Different Visits.

**Figure S4.** Distribution of Global Cognition Z-Score at Different Visits.

**Figure S5.** Linear Regression Analysis For GFAP and NfL Expression with Global Cognition.

**Figure S6.** Comparison of Peripheral GFAP and NfL Expression Level According to the Diagnosis of Dementia.

**Figure S7.** Comparison of Peripheral GFAP and NfL Expression Level According to the Diagnosis of Dementia after Propensity Score Matching.

**Figure S8.** Comparison of Baseline Cognitive Measurements Between Never and Potential Dementia Groups after Propensity Score Matching.

**Figure S9.** Correlation Between Time to a Dementia Diagnosis with Baseline Peripheral GFAP and NfL.

**Figure S10.** Trajectories of GFAP Expression Over 15 Years Preceding Diagnosis of Dementia Using Loess Regression.

**Figure S11.** Trajectories of NfL Expression Over 15 Years Preceding Diagnosis of Dementia Using Loess Regression.

## Tables S1-S16

**Table S1.** Field IDs Used in Analysis.

**Table S2.** Data Categorization.

**Table S3.** Missingness at Baseline for the Cohort.

**Table S4.** Baseline Demographic Characteristics Among Individuals Enrolled in UK Biobank and Evaluated for Olink’s Assay and Cognition.

**Table S5.** Baseline Characteristics Grouped by GFAP Quartile.

**Table S6.** Baseline Characteristics Grouped by NfL Quartile.

**Table S7.** Comparision of Baseline Characteristics Between Participants with Multiple Protein Measurements and Single Protein Measurement.

**Table S8.** Hazard Ratios for All-cause Dementia According to GFAP and NfL Quartiles.

**Table S9.** Correlation Between GFAP and NfL with Age.

**Table S10.** Association Between Annualized Change Rate of GFAP and NfL with Annualized Change Rate of Global Cognition.

**Table S11.** Association Between Annualized Change Rate of GFAP and NfL with Annualized Change Rate of Global Cognition (Setting Follow-up Time as Random Slope).

**Table S12.** Values of Predictive Models Under Leave-One-Region-Out Validations.

**Table S13.** Competing Risk Analysis.

**Table S14.** Sensitivity Analysis for the Association Between GFAP and NfL With Incident All-cause Dementia (Imputing Missing Values).

**Table S15.** Predictive Model Using Age or Protein Expressions and Stratified by Age Groups.

**Table S16.** Predictive Model Using DRSm Combined with Protein Expression and Stratified by Age Groups.

**Supplementary Methods**

We divided participants into three groups based on the dementia diagnosis. The “Never” group means no dementia record in the cohort; the “Potential” group means dementia diagnosis was made after recruitment; the “Existing” group means dementia diagnosed has already been made at recruitment. The Existing group was excluded for analysis in Cox proportional hazard models.

Propensity score matching (PSM) was performed using the MatchIt package (version 4.5.3). Age and sex were matched for the Never group and Potential group with a 1:10 ratio. Specifically, we employed a logistic regression model for propensity score calculation, where age and gender were the independent variables. This model estimated the likelihood of being in the group with potential dementia.

The unpaired Student's t-test assessed differences in baseline GFAP and NfL NPX between each two groups, as well as the cognitive measurements. Pearson's correlation evaluated the association between time to a dementia diagnosis made and age with baseline protein NPX levels.

Annualized change rate of NPX levels and global cognition were calculated using the following formula:

$$Annualized change rate=\frac{Value at later visit-Value at earlier visit}{Time between two visits in years}$$

Annualized change rates from instance 0 to 2, 0 to 3, and 2 to 3 were calculated respectively. Generalized linear mixed models (GLMM) from the lme4 package (version 1.1-34) and lmerTest package (version 3.1-3) were employed to examine the relationship between annualized change rate of GFAP and NfL NPX with global cognition, adjusting for the three confounder models in the Methods section. All mixed models incorporated a random intercept per individual and a fixed slope:

lmer(dglobal_cognition ~ age + age_squared + bmi + sex + ...(other covariates) + dgfap*fu_time + dnefl*fu_time + (1 | participant_id), data = d_data)

For sensitivity analysis, follow-up time as employed as the random slope:

lmer(dglobal_cognition ~ age + age_squared + bmi + sex + ...(other covariates) + dgfap*fu_time + dnefl*fu_time + (1 + fu_time | participant_id), data = d_data)

For competing risk analysis, we first identified the number of non-dementia, non-Alzheimer's disease deaths in our study population, which totaled 4,935 individuals. We then conducted a competing risk analysis using the “cmprsk” package in R. This analysis considered dementia diagnosis as the event of interest (coded as 1) and non-dementia, non-Alzheimer's disease death as the competing event (coded as 2). Our model included the same covariates as in Model 3 in the Method section. The results of this competing risk analysis indicate that individuals with GFAP and NfL levels in the Interval 4 continue to show an increased risk of dementia. **Figure S1.** Distribution of Age at Dementia Diagnosis.


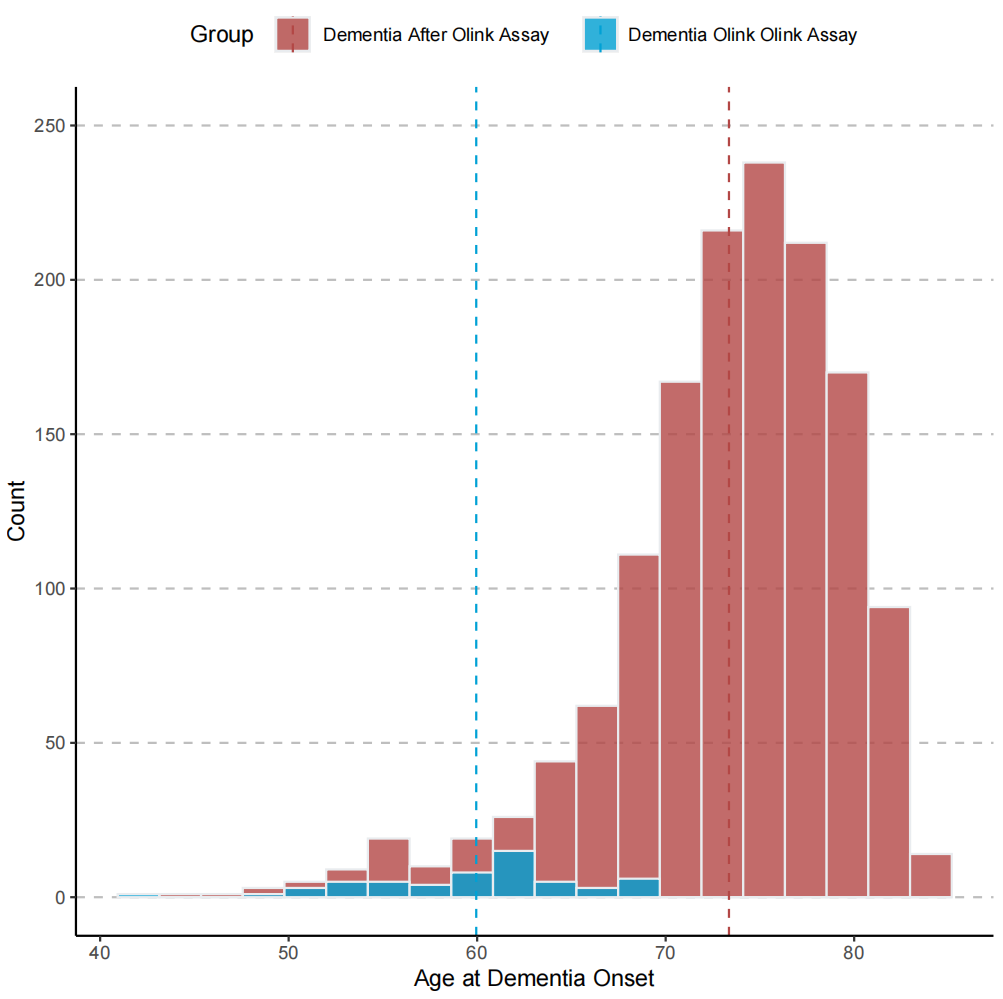
.

The vertical dot lines indicate mean ages for dementia diagnosis.

**Figure S2.** Distribution of GFAP and NfL Expression at Different Visits.


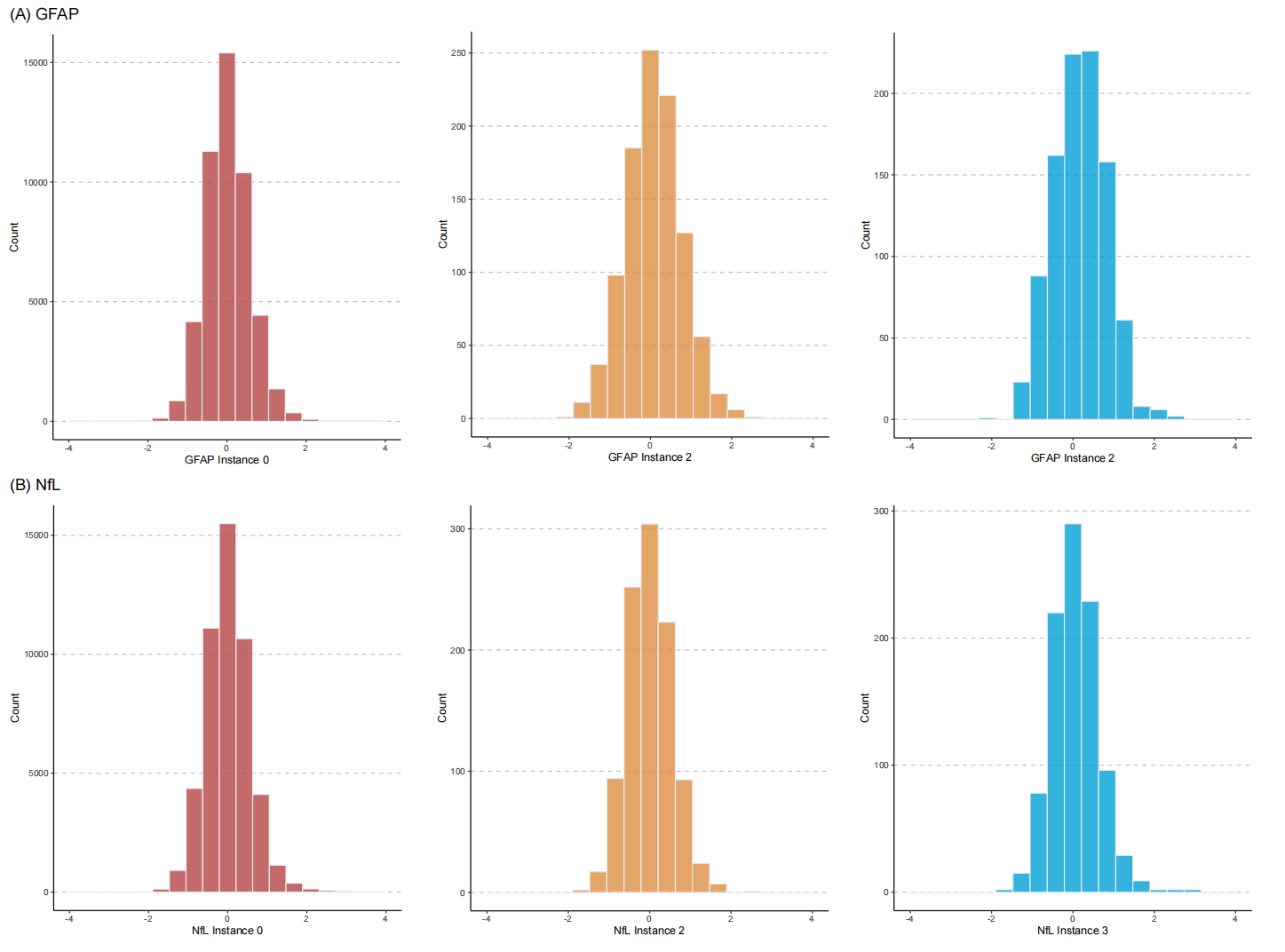


**Figure S3.** Distribution of Cognitive Measurements at Different Visits.


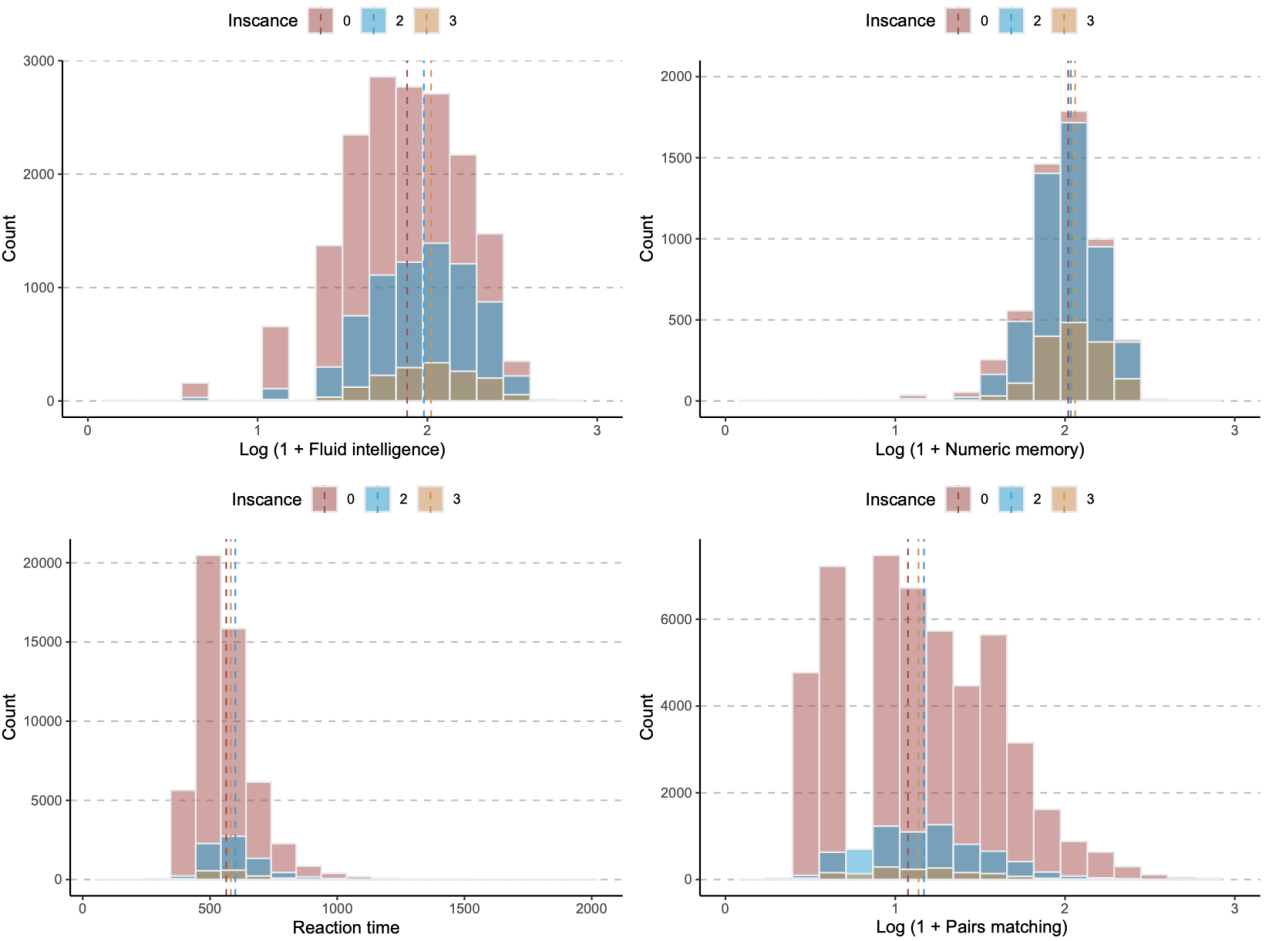


The vertical dot lines indicate mean values for cognitive measurement at different visits.

**Figure S4.** Distribution of Global Cognition Z-Score at Different Visits.


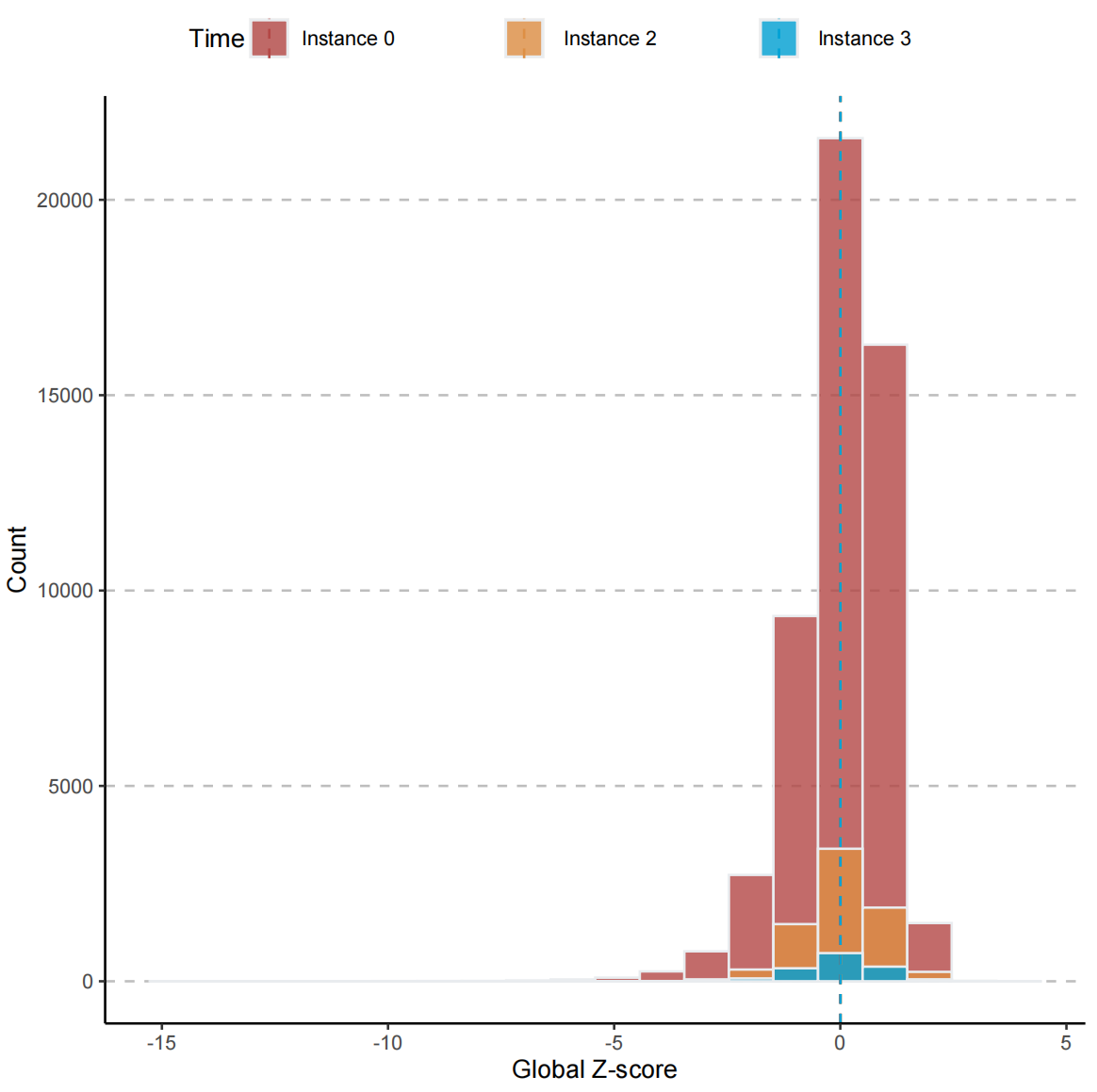


The vertical dot lines indicate mean values for Z-score at different visits.

**Figure S5.** Linear Regression Analysis For GFAP and NfL Expression with Global Cognition.


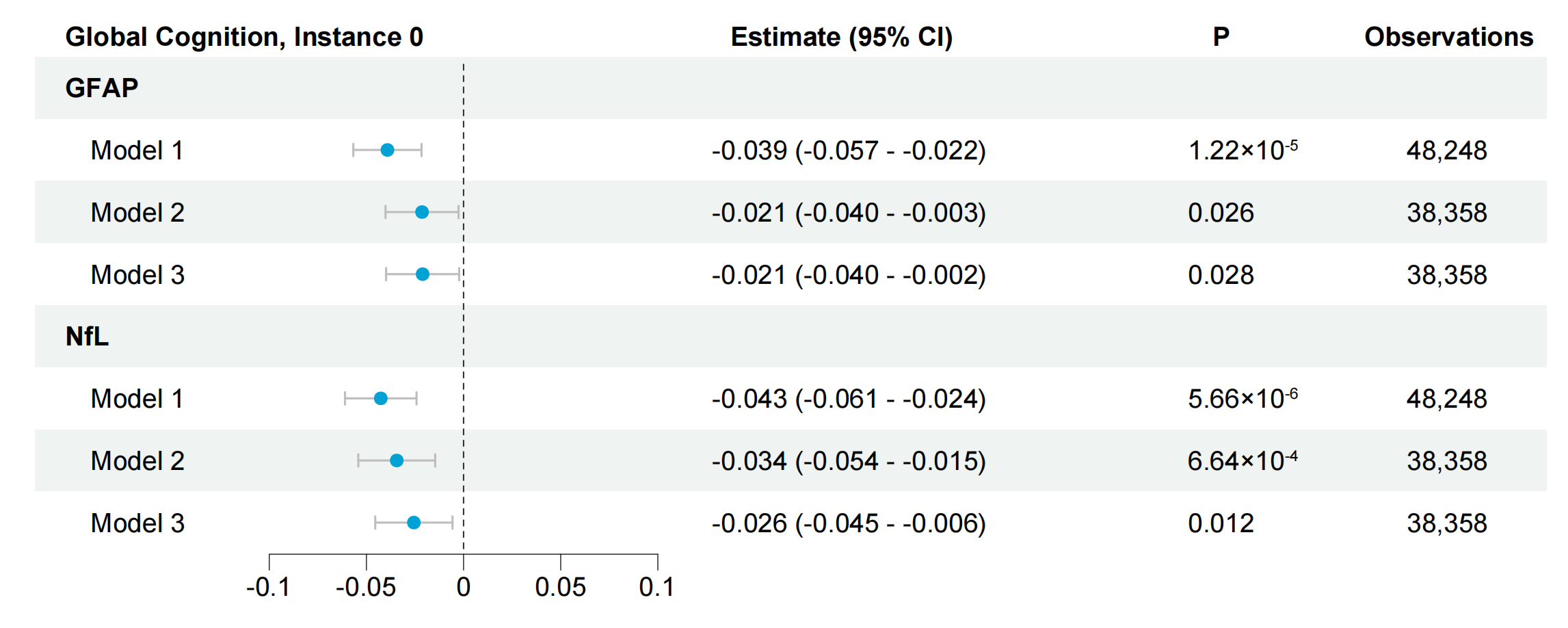


**Figure S6.** Comparison of Peripheral GFAP and NfL Expression Level According to the Diagnosis of Dementia.


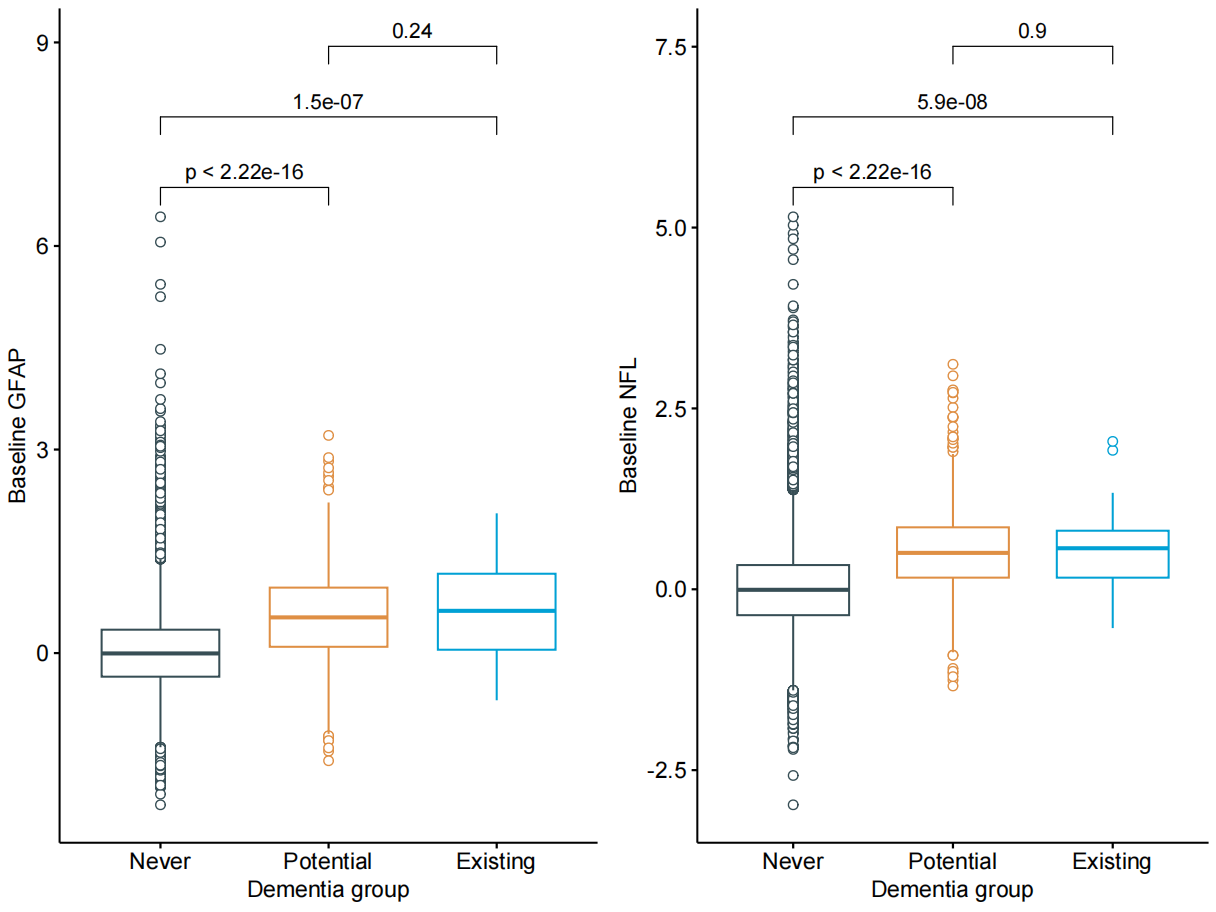


Mean (SD) age: Never, 56.59 ± 8.18 years; Potential, 64.61 ± 5.04 years; Existing, 63.23 ± 5.76 years.

Sample Size: Existing, 48; Potential, 1,312; Never, 47,182.

**Figure S7.** Comparison of Peripheral GFAP and NfL Expression Level According to the Diagnosis of Dementia after Propensity Score Matching.


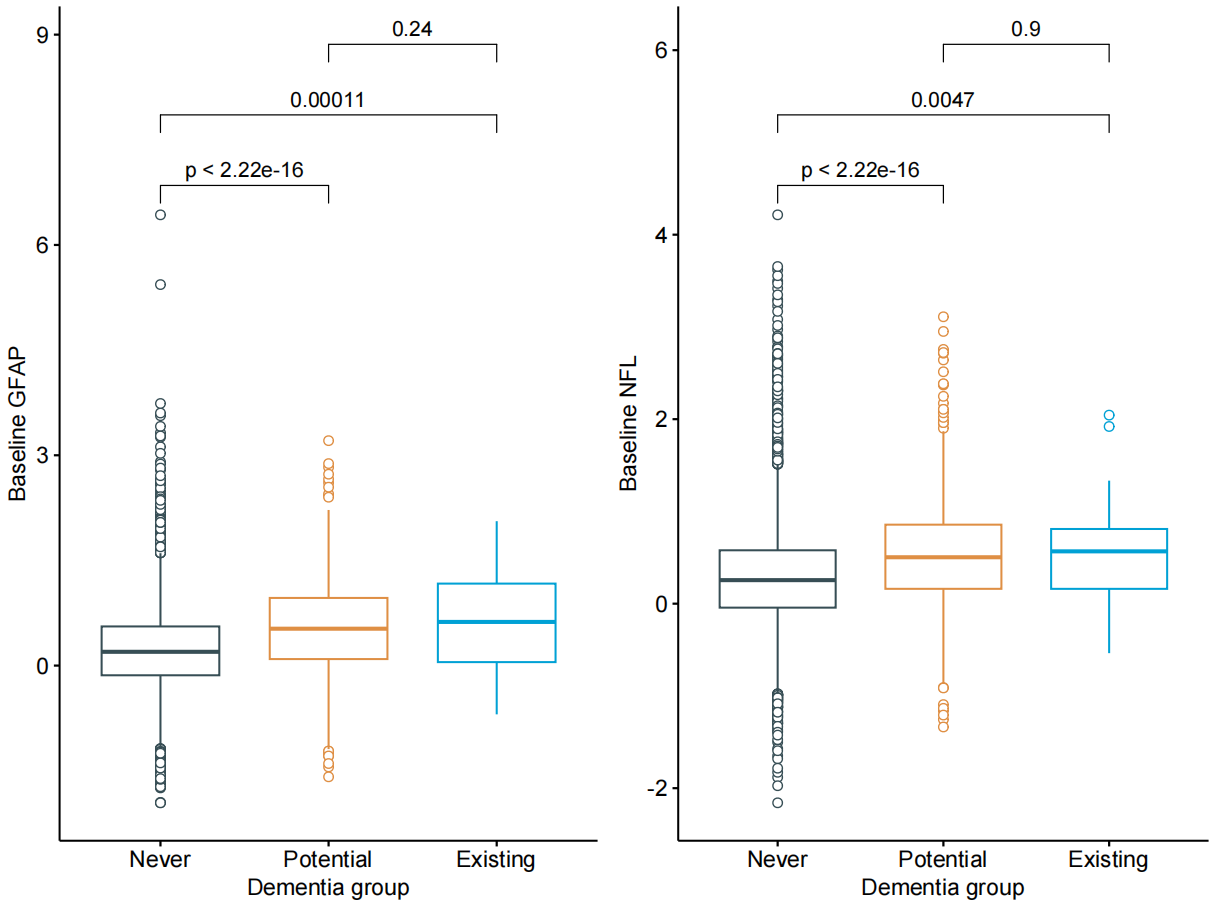


Mean (SD) age: Never, 64.43 ± 4.92 years; Potential, 64.61 ± 5.04 years; Existing, 63.23 ± 5.76 years.

Sample Size: Existing, 48; Potential, 1,312; Never, 13,120.

**Figure S8.** Comparison of Baseline Cognitive Measurements Between Never and Potential Dementia Groups after Propensity Score Matching.


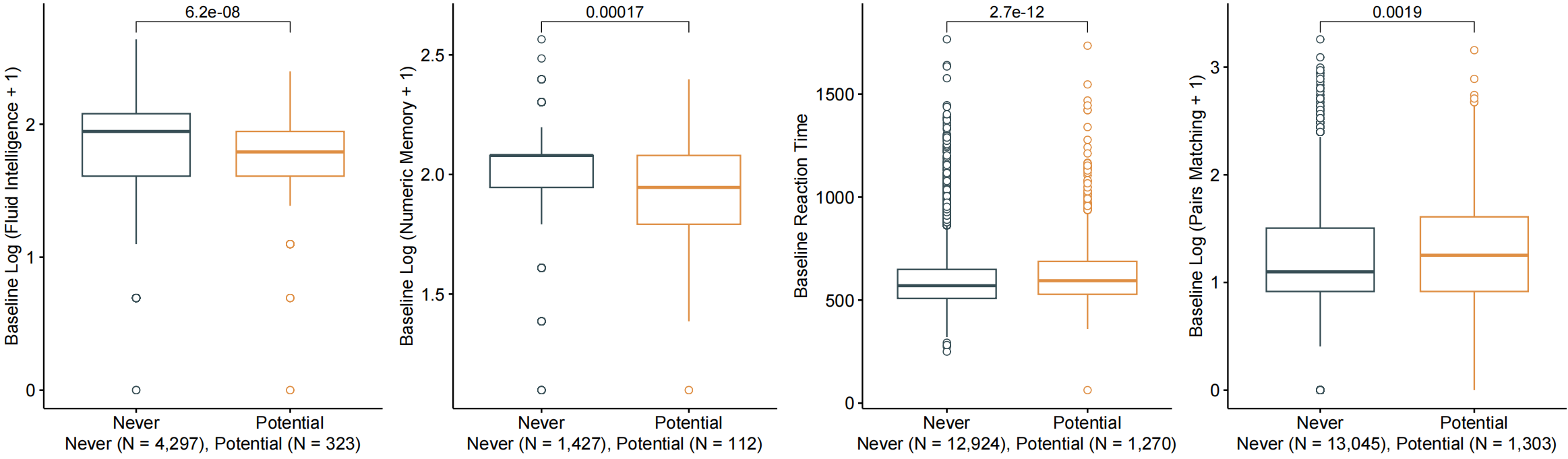


**Figure S9.** Correlation Between Time to a Dementia Diagnosis with Baseline Peripheral GFAP and NfL.


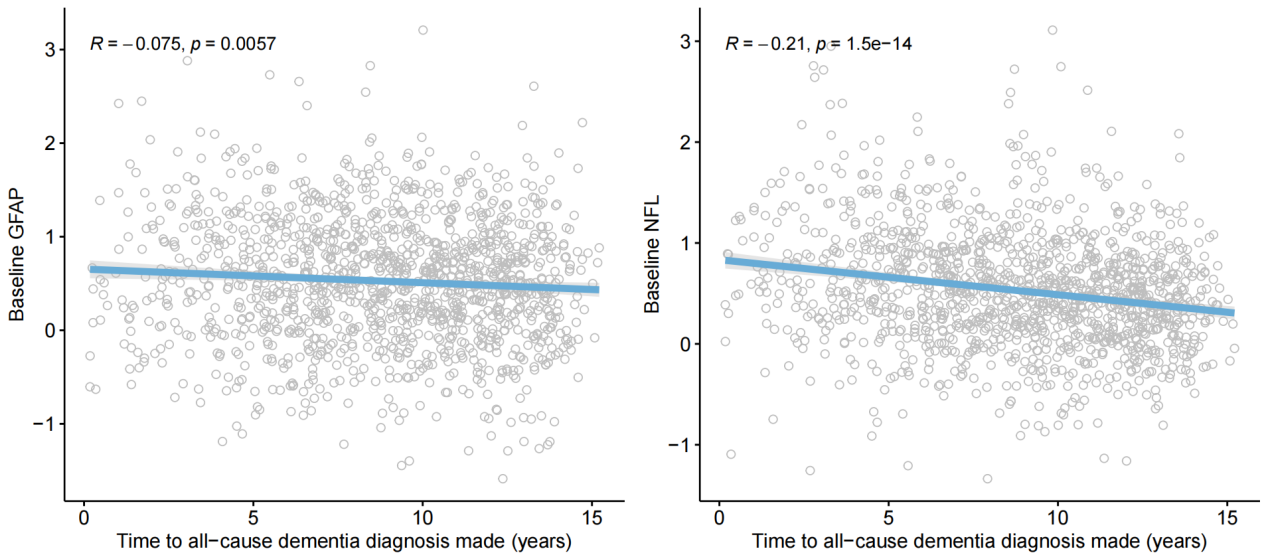


**Figure S10.** Trajectories of GFAP Expression Over 15 Years Preceding Diagnosis of Dementia Using Loess Regression.


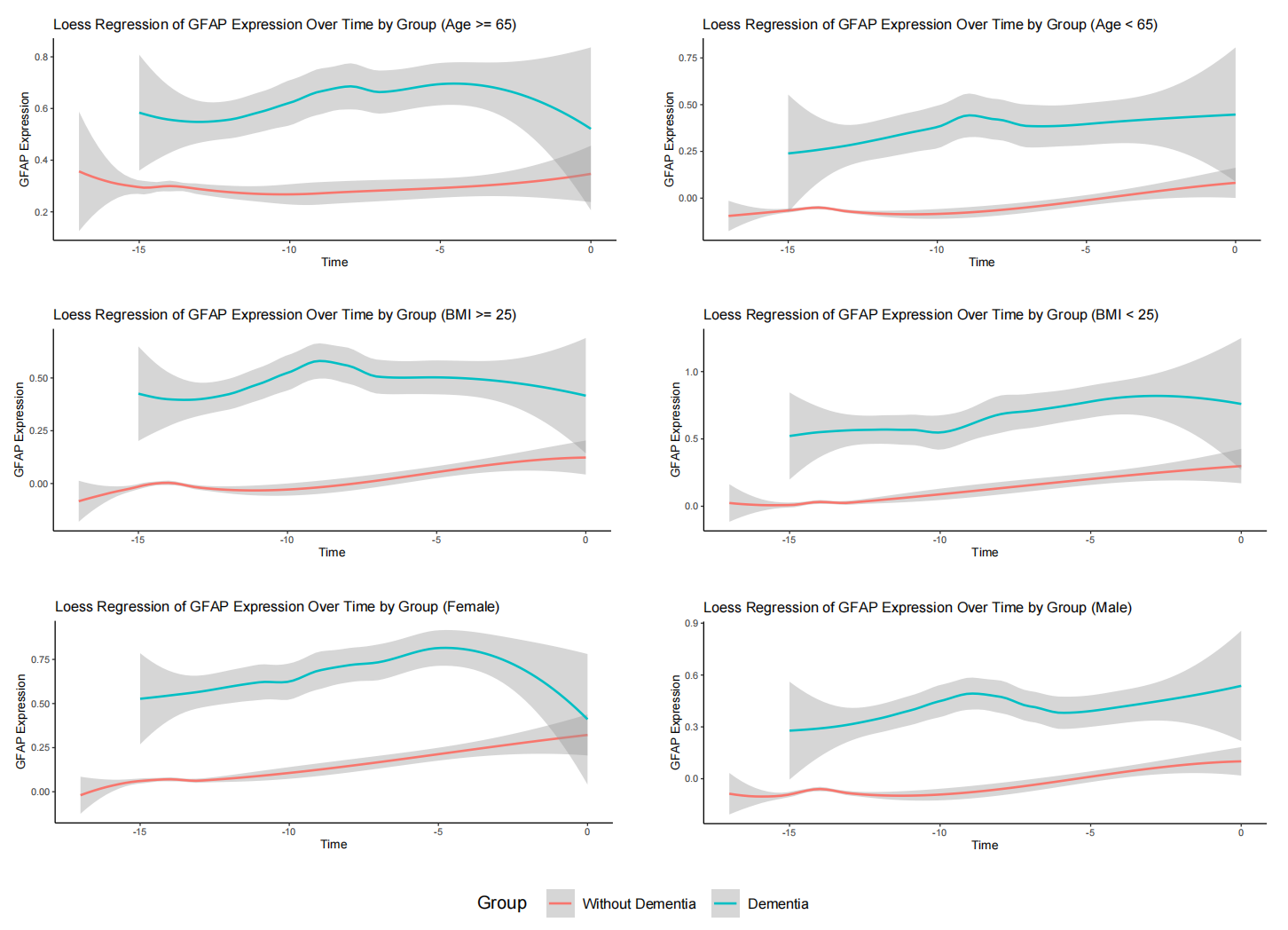


**Figure S11.** Trajectories of NfL Expression Over 15 Years Preceding Diagnosis of Dementia Using Loess Regression.


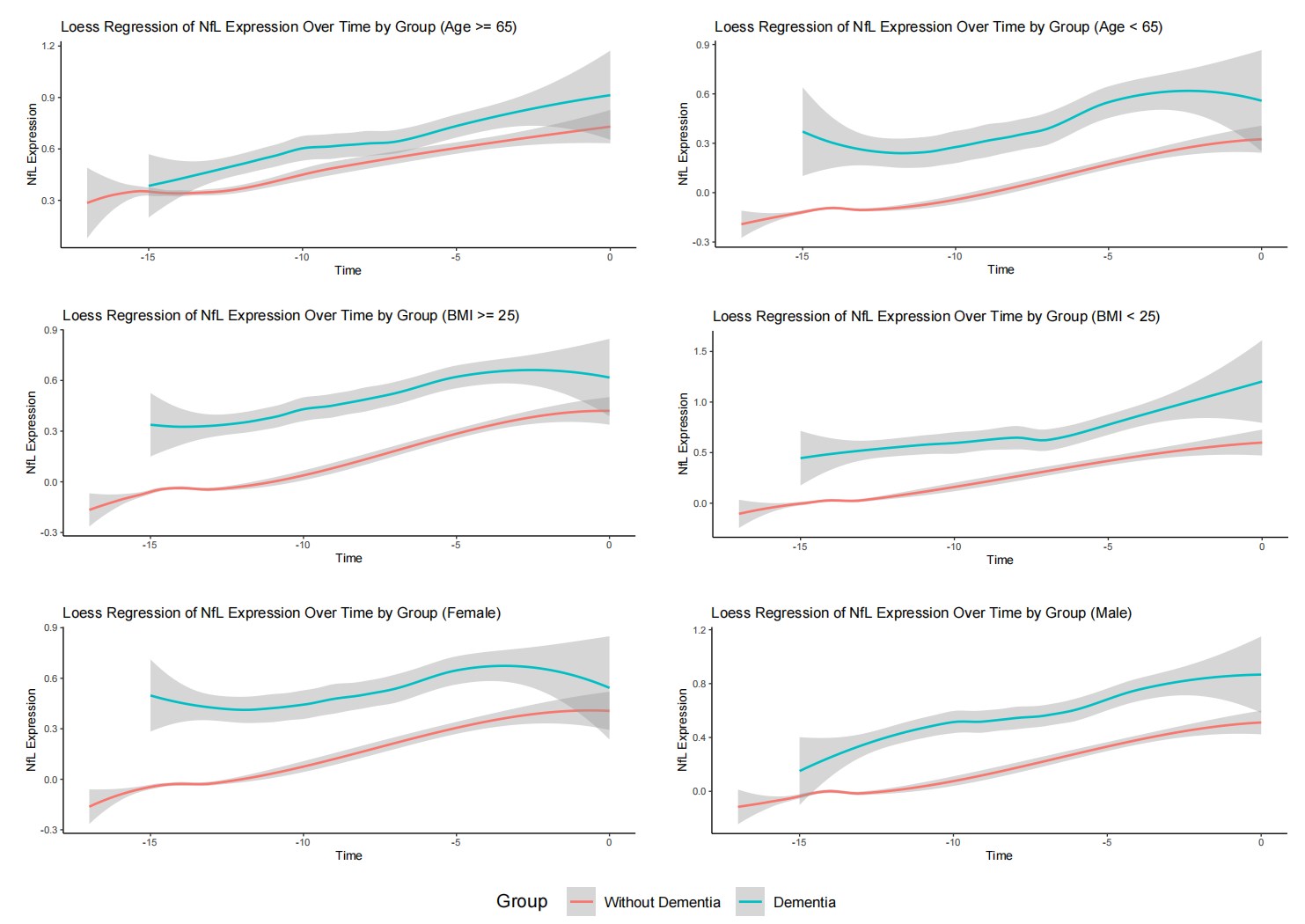


**Table S1.** Field IDs Used in Analysis.

| **Field ID** | **Description** |
| --- | --- |
| eid | Participant ID |
| 31 | Sex |
| 54 | UK Biobank assessment centre |
| 21003 | Age when attended assessment centre |
| 53 | Date of attending assessment centre |
| 21001 | Body mass index (BMI) |
| 6138 | Qualifications |
| 22189 | Townsend deprivation index at recruitment |
| 884 | Days per week of moderate physical activity |
| 20116 | Smoking status |
| 1558 | Alcohol frequency |
| 21000 | Ethnic background |
| 22418 | Genotype |
| 26206 | Standard PRS for alzheimer's disease (AD) |
| **Total cholesterol** | |
| 30690 | Cholesterol in mmol/L |
| **Medication** | |
| 6153 | Medication for cholesterol, blood pressure, diabetes, or take exogenous hormones |
| 20003 | Treatment/medication code |
| **Death cause** | |
| 40001 | Underlying (primary) cause of death: ICD10 |
| cause_icd10 | Cause of death - ICD-10 |
| **Depressive disorders** | |
| 130894 | Date F32 first reported (depressive episode) |
| 130896 | Date F33 first reported (recurrent depressive disorder) |
| **Atrial fibrillation** | |
| 131350 | Date I48 first reported (atrial fibrillation and flutter) |
| **Diabetes** | |
| 130706 | Date E10 first reported (insulin-dependent diabetes mellitus) |
| 130708 | Date E11 first reported (non-insulin-dependent diabetes mellitus) |
| 130710 | Date E12 first reported (malnutrition-related diabetes mellitus) |
| 130712 | Date E13 first reported (other specified diabetes mellitus) |
| 130714 | Date E14 first reported (unspecified diabetes mellitus) |
| **Hypertension** | |
| 131286 | Date I10 first reported (essential (primary) hypertension) |
| 131294 | Date I15 first reported (secondary hypertension) |
| **Cerebrovascular disorders** | |
| 131360 | Date I60 first reported (subarachnoid haemorrhage) |
| 131362 | Date I61 first reported (intracerebral haemorrhage) |
| 131364 | Date I62 first reported (other nontraumatic intracranial haemorrhage) |
| 131366 | Date I63 first reported (cerebral infarction) |
| 131368 | Date I64 first reported (stroke, not specified as haemorrhage or infarction) |
| 131370 | Date I65 first reported (occlusion and stenosis of precerebral arteries, not resulting in cerebral infarction) |
| 131372 | Date I66 first reported (occlusion and stenosis of cerebral arteries, not resulting in cerebral infarction) |
| 131374 | Date I67 first reported (other cerebrovascular diseases) |
| 131376 | Date I68 first reported (cerebrovascular disorders in diseases classified elsewhere) |
| 131056 | Date G45 first reported (transient cerebral ischaemic attacks and related syndromes) |
| 131058 | Date G46 first reported (vascular syndromes of brain in cerebrovascular diseases) |
| **Demyelinating disorders** | |
| 131042 | Date G35 first reported (multiple sclerosis) |
| 131044 | Date G36 first reported (other acute disseminated demyelination) |
| 131046 | Date G37 first reported (other demyelinating diseases of central nervous system) |
| **Neurodegenerative disorders** | |
| 131022 | Date G20 first reported (parkinson's disease) |
| 131024 | Date G21 first reported (secondary parkinsonism) |
| 131026 | Date G22 first reported (parkinsonism in diseases classified elsewhere) |
| 131028 | Date G23 first reported (other degenerative diseases of basal ganglia) |
| 131032 | Date G25 first reported (other extrapyramidal and movement disorders) |
| 131038 | Date G31 first reported (other degenerative diseases of nervous system, not elsewhere classified) |
| 131040 | Date G32 first reported (other degenerative disorders of nervous system in diseases classified elsewhere) |
| 131012 | Date G10 first reported (huntington's disease) |
| 131014 | Date G11 first reported (hereditary ataxia) |
| 131016 | Date G12 first reported (spinal muscular atrophy and related syndromes) |
| **Mental disorders** | |
| 130874 | Date F20 first reported (schizophrenia) |
| 130876 | Date F21 first reported (schizotypal disorder) |
| 130878 | Date F22 first reported (persistent delusional disorders) |
| 130880 | Date F23 first reported (acute and transient psychotic disorders) |
| 130882 | Date F24 first reported (induced delusional disorder) |
| 130884 | Date F25 first reported (schizoaffective disorders) |
| 130886 | Date F28 first reported (other nonorganic psychotic disorders) |
| 130888 | Date F29 first reported (unspecified nonorganic psychosis) |
| 130890 | Date F30 first reported (manic episode) |
| 130892 | Date F31 first reported (bipolar affective disorder) |
| 130894 | Date F32 first reported (depressive episode) |
| 130896 | Date F33 first reported (recurrent depressive disorder) |
| 130898 | Date F34 first reported (persistent mood [affective] disorders) |
| 130900 | Date F38 first reported (other mood [affective] disorders) |
| 130902 | Date F39 first reported (unspecified mood [affective] disorder) |
| 130904 | Date F40 first reported (phobic anxiety disorders) |
| 130907 | Date F41 first reported (other anxiety disorders) |
| 130854 | Date F10 first reported (mental and behavioural disorders due to use of alcohol) |
| 130856 | Date F11 first reported (mental and behavioural disorders due to use of opioids) |
| 130858 | Date F12 first reported (mental and behavioural disorders due to use of cannabinoids) |
| 130860 | Date F13 first reported (mental and behavioural disorders due to use of sedatives or hypnotics) |
| 130862 | Date F14 first reported (mental and behavioural disorders due to use of cocaine) |
| 130864 | Date F15 first reported (mental and behavioural disorders due to use of other stimulants, including caffeine) |
| 130866 | Date F16 first reported (mental and behavioural disorders due to use of hallucinogens) |
| 130868 | Date F17 first reported (mental and behavioural disorders due to use of tobacco) |
| 130870 | Date F18 first reported (mental and behavioural disorders due to use of volatile solvents) |
| 130872 | Date F19 first reported (mental and behavioural disorders due to multiple drug use and use of other psychoactive substances) |
| **Organic brain disorders** | |
| 130844 | Date F04 first reported (organic amnesic syndrome, not induced by alcohol and other psychoactive substances) |
| 130846 | Date F05 first reported (delirium, not induced by alcohol and other psychoactive substances) |
| 130848 | Date F06 first reported (other mental disorders due to brain damage and dysfunction and to physical disease) |
| 130850 | Date F07 first reported (personality and behavioural disorders due to brain disease, damage and dysfunction) |
| 130852 | Date F09 first reported (unspecified organic or symptomatic mental disorder) |
| 131110 | Date G91 first reported (hydrocephalus) |
| 131112 | Date G92 first reported (toxic encephalopathy) |
| 131114 | Date G93 first reported (other disorders of brain) |
| 131116 | Date G94 first reported (other disorders of brain in diseases classified elsewhere) |
| **Dementia** | |
| 131036 | Date G30 first reported (alzheimer's disease) |
| 130836 | Date F00 first reported (dementia in alzheimer's disease) |
| 130838 | Date F01 first reported (vascular dementia) |
| 130840 | Date F02 first reported (dementia in other diseases classified elsewhere) |
| 130842 | Date F03 first reported (unspecified dementia) |
| 42018 | Date of all cause dementia report |
| 42020 | Date of alzheimer's disease report |
| 42022 | Date of vascular dementia report |
| 42024 | Date of frontotemporal dementia report |
| **Cognition** | |
| 20016 | Fluid intelligence score |
| 4282 | Maximum digits remembered correctly (Numeric memory) |
| 20023 | Mean time to correctly identify matches (Reaction time) |
| 399 | Number of incorrect matches in round (Pairs matching) |

**Table S2.** Data Categorization.

| **Variate** | **Categorization or value** |
| --- | --- |
| **Smoking status** | |
| Never | 1 |
| Current, Previous | 2 |
| Prefer not to answer, Missing value | 2 |
| **Alcohol frequency** | |
| Never | 0 |
| Special occasions only | 1 |
| One to three times a month | 2 |
| Once or twice a week | 3 |
| Three or four times a week | 4 |
| Daily or almost daily | 5 |
| **Qualifications** | |
| College or University degree, A levels/AS levels or equivalent, NVQ or HND or HNC or equivalent, Other professional qualifications | 2 |
| Other educational levels, Prefer not to answer, Missing value | 1 |
| **Ethnic background** | |
| British, White, Irish, Any other white background | White |
| Black or Black British, White and Black Caribbean, White and Black African, African, Caribbean, Any other Black background | Black |
| Asian or Asian British, Chinese, Indian, Pakistani, Bangladeshi, Any other Asian background | Asian |
| Mixed, Any other mixed background, Other ethnic group | Multiracial |
| **Dementia diagnosis** | |
| p131036, 130836, p42020 | Alzheimer’s disease and related dementia |
| p130838, p42022 | Vascular dementia |
| p42024 | Frontotemporal dementia |
| p130840, p130842, p42018, and the above data-field | All-cause dementia |

**Table S3.** Missingness at Baseline for the Cohort.

|  | **Total (N = 48,542)** |
| --- | --- |
| Missing BMI | 240 (0.5%) |
| Missing Townsend Deprivation Index | 59 (0.1%) |
| Missing Physical Activity | 2,697 (5.6%) |
| Missing Alcohol frequency | 117 (0.2%) |
| Missing Ethnic background | 236 (0.5%) |
| Missing APOE*E4 Genotype | 7,656 (15.8%) |
| Missing Genetic risk score for AD | 514 (1.1%) |

Abbreviations: BMI = body mass index (kg/m2); APOE = apolipoprotein; AD = Alzheimer’s disease.

**Table S4.** Baseline Demographic Characteristics Among Individuals Enrolled in UK Biobank and Evaluated for Olink’s Assay and Cognition.

|  | **Fluid intelligence**  **(N = 16,906)** | **Numeric memory**  **(N = 5,536)** | **Reaction time**  **(N = 52,055)** | **Pairs matching**  **(N = 52,404)** |
| --- | --- | --- | --- | --- |
| Follow-up time, mean (SD) | 12.4 (1.95) | 12.69 (2.08) | 13.2 (2.35) | 13.18 (2.36) |
| Sex (Female), n (%) | 9,117 (53.9) | 2,990 (54.0) | 28,094 (54.0) | 28,257 (53.9) |
| Age in yeas, mean (SD) | 56.8 (8.24) | 56.8 (8.38) | 56.8 (8.21) | 56.8 (8.21) |
| BMI, mean (SD) | 27.4 (4.79) | 27.5 (4.83) | 27.5 (4.80) | 27.5 (4.80) |
| GFAP NPX, median [min, max] | -0.012 [-2.24, 5.43] | 0.002 [-2.08, 4.48] | 0 [-2.24, 6.43] | 0 [-2.24, 6,43] |
| NfL NPX, median [min, max] | -0.006 [-2.07, 5.15] | 0.002 [-2.08, 4.48] | 0 [-2.98, 5.15] | 0 [-2.98, 5.15] |
| Townsend Deprivation Index,  median [min, max] | -1.79 [-6.26, 9.89] | -2.15 [-6.26, 9.89] | -2.07 [-6.26, 10.4] | -2.05 [-6.26, 10.4] |
| High school equivalent or more schooling, n (%) | 11,476 (67.9) | 3,621 (65.4) | 33,689 (64.7) | 33,905 (64.7) |
| Days per week of moderate physical activity, mean (SD) | 3.72 (2.32) | 3.80 (2.34) | 3.62 (2.34) | 3.62 (2.34) |
| Former or current smoker, n (%) | 7,710 (45.6) | 2,546 (46.0) | 23,892 (45.9) | 24,021 (45.8) |
| Alcohol frequency, n (%) |  |  |  |  |
| Never, n (%) | 1,479 (8.7) | 484 (8.7) | 4,380 (8.4) | 4,536 (8.7) |
| Special occasions only, n (%) | 2,049 (12.1) | 641 (11.6) | 6,096 (11.7) | 6,166 (11.8) |
| One to three times a month, n (%) | 1,906 (11.3) | 639 (11.5) | 5,692 (10.9) | 5,714 (10.9) |
| Once or twice a week, n (%) | 4,235 (25.1) | 1,376 (24.9) | 13,526 (26.0) | 13,576 (25.9) |
| Three or four times a week, n (%) | 3,762 (22.3) | 1,242 (22.4) | 11,757 (22.6) | 11,774 (22.5) |
| Daily or almost daily, n (%) | 3,464 (20.5) | 1,151 (20.8) | 10,552 (20.3) | 10,576 (20.2) |
| Race |  |  |  |  |
| White, n (%) | 15,371 (90.9) | 5,226 (94.4) | 48,799 (93.7) | 48,929 (93.4) |
| Black, n (%) | 604 (3.6) | 76 (1.4) | 1,264 (2.4) | 1,340 (2.6) |
| Asian, n (%) | 558 (3.3) | 155 (2.8) | 1,127 (2.2) | 1,215 (2.3) |
| Multiracial, n (%) | 308 (1.8) | 65 (1.2) | 682 (1.3) | 727 (1.4) |
| APOE*E4 carrier, n (%) |  |  |  |  |
| E2E4 & E3E4 | 3,687 (21.8) | 1,197 (21.6) | 11,474 (22.0) | 11,598 (22.1) |
| E4E4 | 416 (2.5) | 141 (2.5) | 1,268 (2.4) | 1,283 (2.4) |
| Comorbidity, n (%) |  |  |  |  |
| Diabetes, n (%) | 957 (5.7) | 305 (5.5) | 2,905 (5.6) | 2,973 (5.7) |
| Hypertension, n (%) | 4,684 (27.7) | 1,529 (27.6) | 14,582 (28.0) | 14,741 (28.1) |
| Cerebrovacular disorders, n (%) | 421 (2.5) | 135 (2.4) | 1,300 (2.5) | 1,324 (2.5) |
| Demyelinating disorders, n (%) | 125 (0.7) | 48 (0.9) | 405 (0.8) | 408 (0.8) |
| Neurodegenerative disorders, n (%) | 185 (1.1) | 64 (1.2) | 505 (1.0) | 510 (1.0) |
| Organic brain diseases, n (%) | 154 (0.9) | 60 (1.1) | 499 (1.0) | 509 (1.0) |
| Mental disorders, n (%) | 2,952 (17.5) | 1,196 (21.6) | 8,298 (15.9) | 8,388 (16.0) |

Abbreviations: BMI, body mass index (kg/m2); NPX, normalized protein expression; APOE, apolipoprotein.

**Table S5.** Baseline Characteristics Grouped by GFAP Quartile.

|  | **GFAP Interval 1**  **(N = 12,136)** | **GFAP Interval 2**  **(N = 12,309)** | **GFAP Interval 3**  **(N = 11,961)** | **GFAP Interval 4**  **(N = 12,136)** |
| --- | --- | --- | --- | --- |
| GFAP NPX, median [min, max] | -0.583 [-2.24, -0.341] | -0.157 [-0.341, 0] | 0.167 [0, 0.363] | 0.646 [0.363, 6.43] |
| Sex (Female), n (%) | 5,581 (46.0) | 6,263 (50.9） | 6,646 (55.6) | 7,662 (63.1) |
| Age in yeas, mean (SD) | 52.8 (8.07) | 55.3 (8.01) | 57.9 (7.62) | 61.2 (6.63) |
| BMI in kg/m^2^, mean (SD) | 27.9 (5.14) | 27.6 (4.85) | 27.3 (4.60) | 27.1 (4.52) |
| Townsend Deprivation Index, median [min, max] | -1.79 [-6.26, 10.2] | -2.02 [-6.26, 10.4] | -2.15 [-6.26, 10.1] | -2.21 [-6.26, 9.98] |
| High school equivalent or more schooling, n (%) | 8,358 (68.9) | 8,178 (66.4) | 7,666 (64.1) | 7,029 (57.9) |
| Days per week of moderate physical activity, mean (SD) | 3.56 (2.33) | 3.57 (2.33) | 3.65 (2.34) | 3.69 (2.36) |
| Former or current smoker, n (%) | 5,692 (46.9) | 5,741 (46.6) | 5,397 (45.1) | 5,409 (44.6) |
| Alcohol frequency, n (%) |  |  |  |  |
| Never, n (%) | 943 (7.8) | 1,018 (8.3) | 986 (8.2) | 1,251 (10.3) |
| Special occasions only, n (%) | 1,338 (11.0) | 1,321 (10.7) | 1,377 (11.5) | 1,656 (13.6) |
| One to three times a month, n (%) | 1,343 (11.1) | 1,299 (10.6) | 1,278 (10.7) | 1,336 (11.0) |
| Once or twice a week, n (%) | 3,236 (26.7) | 3,244 (26.4) | 3,106 (26.0) | 3,010 (24.8) |
| Three or four times a week, n (%) | 2,836 (23.4) | 2,917 (23.7) | 2,681 (22.4) | 2,467 (20.3) |
| Daily or almost daily, n (%) | 2,408 (19.8) | 2,484 (20.2) | 2,503 (20.9) | 2,387 (19.7) |
| Race |  |  |  |  |
| White, n (%) | 11,174 (92.1) | 11,421 (92.8) | 11,186 (93.5) | 11,488 (94.7) |
| Black, n (%) | 389 (3.2) | 348 (2.8) | 291 (2.4) | 224 (1.8) |
| Asian, n (%) | 321 (2.6) | 272 (2.2) | 267 (2.2) | 244 (2.0) |
| Multiracial, n (%) | 191 (1.6) | 209 (1.7) | 153 (1.3) | 128 (1.1) |
| APOE*E4 carrier, n (%) |  |  |  |  |
| E2E4 & E3E4 | 2,504 (20.6) | 2,559 (20.8) | 2,602 (21.8) | 3,017 (24.9) |
| E4E4 | 209 (1.7) | 220 (1.8) | 283 (2.4) | 481 (4.0) |
| Comorbidity, n (%) |  |  |  |  |
| Diabetes, n (%) | 780 (6.4) | 649 (5.3) | 644 (5.4) | 670 (5.5) |
| Hypertension, n (%) | 30,06 (24.8) | 32,93 (26.8) | 34,08 (28.5) | 39,44 (32.5) |
| Cerebrovacular disorders, n (%) | 252 (2.1) | 265 (2.2) | 284 (2.4) | 422 (3.5) |
| Demyelinating disorders, n (%) | 56 (0.5) | 94 (0.8) | 93 (0.8) | 139 (1.1) |
| Neurodegenerative disorders, n (%) | 89 (0.7) | 126 (1.0) | 121 (1.0) | 137 (1.1) |
| Organic brain diseases, n (%) | 117 (1.0) | 117 (1.0) | 134 (1.1) | 116 (1.0) |
| Mental disorders, n (%) | 2,090 (17.2) | 2,018 (16.4) | 1,823 (15.2) | 1,815 (15.0) |

Abbreviations: BMI, body mass index (kg/m2); NPX, normalized protein expression; APOE, apolipoprotein.

**Table S6.** Baseline Characteristics Grouped by NfL Quartile.

|  | **NfL Interval 1**  **(N = 12,138)** | **NfL Interval 2**  **(N = 12,305)** | **NfL Interval 3**  **(N = 11,963)** | **NfL Interval 4**  **(N = 12,136)** |
| --- | --- | --- | --- | --- |
| NfL NPX, median [min, max] | -0.596 [-2.98, -0.349] | -0.161 [-0.348, 0] | 0.162 [0, 0.353] | 0.621 [0.353, 5.15] |
| Sex (Female), n (%) | 6,652 (54.8) | 6,707 (54.5) | 6,439 (53.8) | 6,354 (52.4) |
| Age in yeas, mean (SD) | 50.7 (7.38) | 55.5 (7.61) | 59.0 (6.93) | 62.2 (6.05) |
| BMI in kg/m^2^, mean (SD) | 28.1 (5.21) | 27.5 (4.71) | 27.2 (4.57) | 26.9 (4.58) |
| Townsend Deprivation Index, median [min, max] | -1.75 [-6.26, 10.2] | -2.09 [-6.26, 10.2] | -2.14 [-6.26, 10.4] | -2.21 [-6.26, 9.9.98] |
| High school equivalent or more schooling, n (%) | 8,528 (70.3) | 8,107 (65.9) | 7,569 (63.3) | 7,027 (57.9) |
| Days per week of moderate physical activity, mean (SD) | 3.46 (2.32) | 3.59 (2.32) | 3.67 (2.33) | 375 (2.39) |
| Former or current smoker, n (%) | 5,257 (43.3) | 5,603 (45.5) | 5,542 (46.3) | 5,837 (48.1) |
| Alcohol frequency, n (%) |  |  |  |  |
| Never, n (%) | 1,009 (8.3) | 970 (7.9) | 1,028 (8.6) | 1,191 (9.8) |
| Special occasions only, n (%) | 1,369 (11.3) | 1,313 (10.7) | 1,401 (11.7) | 1,609 (13.3) |
| One to three times a month, n (%) | 1,469 (12.1) | 1,336 (10.9) | 1,188 (9.9) | 1,263 (10.4) |
| Once or twice a week, n (%) | 3,285 (27.1) | 3,205 (260.0) | 3,120 (26.1) | 2,986 (24.6) |
| Three or four times a week, n (%) | 2,839 (2.4) | 2,949 (24.0) | 2,664 (22.3) | 2,449 (20.2) |
| Daily or almost daily, n (%) | 2,135 (17.6) | 2,500 (20.3) | 2,533 (21.2) | 2,614 (21.5) |
| Race |  |  |  |  |
| White, n (%) | 10,885 (89.7) | 11,492 (93.4) | 11,318 (94.6) | 11,574 (95.4) |
| Black, n (%) | 619 (5.1) | 295 (2.4) | 198 (1.7) | 140 (1.2) |
| Asian, n (%) | 326 (2.7) | 265 (2.2) | 253 (2.1) | 260 (2.1) |
| Multiracial, n (%) | 255 (2.1) | 191 (1.6) | 133 (1.1) | 102 (0.8) |
| APOE*E4 carrier, n (%) |  |  |  |  |
| E2E4 & E3E4 | 2,680 (22.1) | 2,604 (21.2) | 2,633 (22.0) | 2,765 (22.8) |
| E4E4 | 232 (1.9) | 256 (2.1) | 295 (2.5) | 410 (3.4) |
| Comorbidity, n (%) |  |  |  |  |
| Diabetes, n (%) | 477 (3.9) | 538 (4.4) | 657 (5.5) | 1,071 (8.8) |
| Hypertension, n (%) | 2,610 (21.5) | 3,070 (24.9) | 3,501 (29.3) | 4,470 (36.8) |
| Cerebrovacular disorders, n (%) | 159 (1.3) | 242 (2.0) | 302 (2.5) | 520 (4.3) |
| Demyelinating disorders, n (%) | 70 (0.6) | 77 (0.6) | 96 (0.8) | 139 (1.1) |
| Neurodegenerative disorders, n (%) | 60 (0.5) | 96 (0.7) | 117 (1.0) | 210 (1.7) |
| Organic brain diseases, n (%) | 135 (1.1) | 116 (0.9) | 101 (0.8) | 132 (1.1) |
| Mental disorders, n (%) | 1,967 (16.2) | 1,977 (16.1) | 1,861 (15.6) | 1,941 (16.0) |

Abbreviations: BMI, body mass index (kg/m2); NPX, normalized protein expression; APOE, apolipoprotein.

**Table S7.** Comparision of Baseline Characteristics Between Participants with Multiple Protein Measurements and Single Protein Measurement.

|  | **Multiple GFAP or NfL Measurements (N = 1,124)** | **Single GFAP and NfL Measurements at Baseline (N = 47,418)** | **P-value** |
| --- | --- | --- | --- |
| Sex (Female), n (%) | 613 (54.5) | 25,539 (53.9) | 0.674 |
| Age in yeas, mean (SD) | 50.2 (7.13） | 57.0 (8.17) | < 0.001 |
| BMI in kg/m^2^, mean (SD) | 26.3 (4.17) | 27.5 (4.80) | < 0.001 |
| GFAP NPX, median [min, max] | -0.256 [-1.91, 1.85] | 0 [-2.24, 6.43] | < 0.001 |
| Nfl NPX, median [min, max] | -0.237 [-2.16, 3.92] | 0 [-2.98, 5.15] | < 0.001 |
| Townsend Deprivation Index, median [min, max] | -2.37 [-6.26, 8.47] | -2.05 [-6.26, 10.4] | < 0.001 |
| High school equivalent or more schooling, n (%) | 927 (82.5) | 30,304 (63.9) | < 0.001 |
| Days per week of moderate physical activity, mean (SD) | 3.51 (2.28) | 3.62 (2.34) | 0.114 |
| Former or current smoker, n (%) | 426 (37.9) | 21,813 (46.0) | < 0.001 |
| Alcohol frequency, n (%) |  |  | < 0.001 |
| Never, n (%) | 42 (3.7) | 4,156 (8.8) |  |
| Special occasions only, n (%) | 102 (9.1) | 5,590 (11.8) |  |
| One to three times a month, n (%) | 126 (11.2) | 5,130 (10.8) |  |
| Once or twice a week, n (%) | 292 (26.0) | 12,304 (25.9) |  |
| Three or four times a week, n (%) | 326 (29.0) | 10,575 (22.3) |  |
| Daily or almost daily, n (%) | 236 (21.0) | 9,546 (20.1) |  |
| Race |  |  | < 0.001 |
| White, n (%) | 1,088 (96.8) | 44,181 (93.2) |  |
| Black, n (%) | 9 (0.8) | 1,243 (2.6) |  |
| Asian, n (%) | 18 (1.6) | 1,086 (2.3) |  |
| Multiracial, n (%) | 9 (0.8) | 672 (1.4) |  |
| APOE*E4 carrier, n (%) |  |  | 0.062 |
| E2E4 & E3E4 | 228 (20.8) | 10,454 (22.0) |  |
| E4E4 | 18 (1.6) | 1,175 (2.5) |  |
| Comorbidity, n (%) |  |  |  |
| Diabetes, n (%) | 24 (2.1) | 2,719 (5.7) | < 0.001 |
| Hypertension, n (%) | 158 (14.1) | 13,493 (28.5) | < 0.001 |
| Cerebrovacular disorders, n (%) | 6 (0.5) | 1,217 (2.6) | < 0.001 |
| Demyelinating disorders, n (%) | 3 (0.3) | 379 (0.8) | 0.068 |
| Neurodegenerative disorders, n (%) | 3 (0.3) | 470 (1.0) | 0.022 |
| Organic brain diseases, n (%) | 13 (1.2) | 471 (1.0) | 0.695 |
| Mental disorders, n (%) | 170 (15.1) | 7,576 (16.0) | 0.465 |
| Cognition |  |  |  |
| Fluid intelligence, log (measurement + 1), mean (SD) | 2.01 (0.293) | 1.88 (0.348) | < 0.001 |
| Numeric memory, log (measurement + 1), mean (SD) | 2.09 (0.18) | 2.02 (0.19) | < 0.001 |
| Pairs matching, log (measurement + 1), mean (SD) | 0.949 (0.466) | 1.08 (0.529) | < 0.001 |
| Reaction time in ms, mean (SD) | 518 (95.2) | 565 (123) | < 0.001 |
| Dementia diagnosis in total |  |  | - |
| All-cause dementia,n (%) | 0 | 1,360 (2.8) |  |
| Alzheimer’s disease and related dementia,n (%) | 0 | 661 (1.4) |  |
| Vascular dementia,n (%) | 0 | 273 (0.6) |  |
| Frontotemporal dementia,n (%) | 0 | 94 (0.2) |  |

Abbreviations: BMI, body mass index (kg/m2); NPX, normalized protein expression; APOE, apolipoprotein; ms = Milliseconds.

**Table S8.** Hazard Ratios for All-cause Dementia According to GFAP and NfL Quartiles.

| **GFAP (All-cause dementia, Model 3)** | **HR (95% CI)** | **P** | **Observations** |
| --- | --- | --- | --- |
| Interval 1 | Reference | - | 38,324 |
| Interval 2 | 1.11 (0.84 – 1.48) | 0.467 |  |
| Interval 3 | 1.19 (0.91 – 1.55) | 0.215 |  |
| Interval 4 | 2.48 (1.94 – 3.17) | 6.28×10-13 |  |
| **NfL (All-cause dementia, Model 3)** | **HR (95% CI)** | **P** | **Observations** |
| Interval 1 | Reference | - | 38,324 |
| Interval 2 | 1.20 (0.83 – 1.73) | 0.324 |  |
| Interval 3 | 1.66 (1.18 – 2.34) | 0.004 |  |
| Interval 4 | 2.83 (2.02 – 3.95) | 1.17×10-9 |  |

Covariates were adjusted by model 3 as described in Methods.

**Table S9.** Correlation Between GFAP and NfL with Age.

|  | **Pearson r** | **95% CI** | **P-value** |
| --- | --- | --- | --- |
| GFAP | 0.377 | 0.370 – 0.385 | <2.2×10^-16^ |
| NfL | 0.501 | 0.494 – 0.508 | <2.2×10^-16^ |

**Table S10.** Association Between Annualized Change Rate of GFAP and NfL with Annualized Change Rate of Global Cognition.

| **Global Cognition** | **Estimate (95% CI)** | **P** | **Observations** |
| --- | --- | --- | --- |
| **Model 1** |  |  | 2,652 |
| GFAP | -0.066 (-0.122 – -0.009) | **0.023*** |  |
| GFAP:Follow-up time | 0.004 (-0.009 – 0.018) | 0.545 |  |
| NfL | 0.000 (-0.086 – 0.087) | 0.998 |  |
| NfL:Follow-up time | 0.002 (-0.017 – 0.021) | 0.861 |  |
| **Model 2** |  |  | 2,219 |
| GFAP | -0.103 (-0.165 – -0.041) | **0.001**** |  |
| GFAP:Follow-up time | 0.008 (-0.079 – 0.109) | 0.319 |  |
| NfL | 0.015 (-0.007 – 0.023) | 0.761 |  |
| NfL:Follow-up time | 0.000 (-0.021 – 0.021) | 0.986 |  |
| **Model 3** |  |  | 2,219 |
| GFAP | -0.101 (-0.163 – -0.040) | **0.001**** |  |
| GFAP:Follow-up time | 0.007 (-0.078 – 0.110) | 0.337 |  |
| NfL | 0.015 (-0.007 – 0.022) | 0.751 |  |
| NfL:Follow-up time | -0.001 (-0.022 – 0.020) | 0.943 |  |

Covariates were adjusted by model 1, 2, and 3 as described in Methods.

*P<0.05, **P<0.01

**Table S11.** Association Between Annualized Change Rate of GFAP and NfL with Annualized Change Rate of Global Cognition (Setting Follow-up Time as Random Slope).

| **Global Cognition** | **Estimate (95% CI)** | **P** | **Observations** |
| --- | --- | --- | --- |
| **Model 1** |  |  | 2,652 |
| GFAP | -0.063 (-0.134 – 0.008) | 0.084 |  |
| GFAP:Follow-up time | 0.003 (-0.158 – 0.062) | 0.640 |  |
| NfL | -0.048 (-0.008 – 0.013) | 0.394 |  |
| NfL:Follow-up time | 0.006 (-0.009 – 0.021) | 0.439 |  |
| **Model 2** |  |  | 2,219 |
| GFAP | -0.104 (-0.182 – -0.027) | **0.009**** |  |
| GFAP:Follow-up time | 0.006 (-0.006 – 0.018) | 0.305 |  |
| NfL | -0.022 (-0.141 – 0.098) | 0.721 |  |
| NfL:Follow-up time | 0.004 (-0.013 – 0.020) | 0.682 |  |
| **Model 3** |  |  | 2,219 |
| GFAP | -0.103 (-0.181 – -0.026) | **0.009**** |  |
| GFAP:Follow-up time | 0.006 (-0.137 – 0.102) | 0.332 |  |
| NfL | -0.018 (-0.006 – 0.017) | 0.769 |  |
| NfL:Follow-up time | 0.002 (-0.015 – 0.019) | 0.791 |  |

Covariates were adjusted by model 1, 2, and 3 as described in Methods.

*P<0.05, **P<0.01

**Table S12.** Values of Predictive Models Under Leave-One-Region-Out Validations.

|  | **Age** | **GFAP + NfL** | **CAIDE** | **CAIDE + GFAP + NfL** | **DRSm** | **DRSm + GFAP + NfL** |
| --- | --- | --- | --- | --- | --- | --- |
| **AUC** |  |  |  |  |  |  |
| All-cause dementia | 0.806 ± 0.031 | 0.785 ± 0.024 | 0.751 ± 0.036 | 0.832 ± 0.030 | 0.848 ± 0.032 | 0.867 ± 0.025 |
| ADRD | 0.817 ± 0.049 | 0.816 ± 0.036 | 0.791 ± 0.052 | 0.864 ± 0.047 | 0.867 ± 0.050 | 0.892 ± 0.044 |
| VD | 0.822 ± 0.048 | 0.781 ± 0.091 | 0.770 ± 0.077 | 0.843 ± 0.083 | 0.871 ± 0.042 | 0.892 ± 0.047 |
| FTD | 0.699 ± 0.039 | 0.809 ± 0.038 | 0.627 ± 0.014 | 0.819 ± 0.027 | 0.690 ± 0.057 | 0.799 ± 0.036 |
| **C-Index** |  |  |  |  |  |  |
| All-cause dementia | 0.805 ± 0.001 | 0.792 ± 0.001 | 0.754 ± 0.002 | 0.837 ± 0.001 | 0.851 ± 0.002 | 0.871 ± 0.001 |
| ADRD | 0.823 ± 0.002 | 0.829 ± 0.002 | 0.798 ± 0.003 | 0.879 ± 0.002 | 0.876 ± 0.002 | 0.904 ± 0.002 |
| VD | 0.830 ± 0.003 | 0.807 ± 0.004 | 0.775 ± 0.004 | 0.861 ± 0.004 | 0.888 ± 0.002 | 0.910 ± 0.002 |
| FTD | 0.703 ± 0.015 | 0.811 ± 0.011 | 0.680 ± 0.009 | 0.842 ± 0.010 | 0.779 ± 0.024 | 0.858 ± 0.010 |
| **Net Reclassification Index** |  |  |  |  |  |  |
| All-cause dementia | 0.088 (0.065 – 0.106) | | 0.173 (0.152 – 0.203) | | 0.135 (0.108 – 0.171) | |
| ADRD | 0.024 (0.010 – 0.039) | | 0.135 (0.103 – 0.170) | | 0.128 (0.089 – 0.171) | |
| VD | 0.012 (-0.001 – 0.034) | | 0.051 (0.019 – 0.093) | | 0.064 (0.033 – 0.128) | |
| FTD | -0.0002 (-0.0003 – -0.00008) | | -0.0002 (-0.0004 – -0.0001) | | -0.0002 (-3.71E-04 – -9.89E-05) | |

ADRD: Alzheimer’s disease and related dementia; VD: Vascular dementia; FTD: Frontotemporal dementia.

CAIDE: Cardiovascular Risk Factors, Aging, and Incidence of Dementia Risk Score, DRSm: Dementia Risk Score modified

**Table S13.** Competing Risk Analysis.

| **Outcome** | **HR (95% CI)** | **P-value** |
| --- | --- | --- |
| **All-cause dementia** |  |  |
| GFAP (Interval 4) | 2.224 (1.937 – 2.555) | < 2.2×10^-16^ |
| NfL (Interval 4) | 1.869 (1.622 – 2.154) | < 2.2×10^-16^ |
| **Alzheimer's disease and related dementia** |  |  |
| GFAP (Interval 4) | 2.980 (2.407 – 3.688) | < 2.2×10^-16^ |
| NfL (Interval 4) | 1.971 (1.611 – 2.411) | 4.40×10^-11^ |
| **Vascular dementia** |  |  |
| GFAP (Interval 4) | 2.201 (1.631 – 2.969) | 2.40×10^-7^ |
| NfL (Interval 4) | 1.925 (1.407 – 2.633) | 4.20×10^-5^ |
| **Frontotemporal dementia** |  |  |
| GFAP (Interval 4) | 3.115 (1.767 – 5.493) | 8.60×10^-5^ |
| NfL (Interval 4) | 4.074 (2.123 – 7.798) | 2.20×10^-5^ |

Covariates were adjusted by model 3 as described in Methods.

**Table S14.** Sensitivity Analysis for the Association Between GFAP and NfL With Incident All-cause Dementia (Imputing Missing Values).

|  | **HR (95% CI)** | **P** | **Observations** |
| --- | --- | --- | --- |
| **GFAP** |  |  |  |
| Model 1 | 2.33 (2.07 – 2.62) | < 2×10^-16^ | 48,494 |
| Model 2 | 2.09 (1.85 – 2.35) | < 2×10^-16^ | 48,494 |
| Model 3 | 2.18 (1.94 – 2.45) | < 2×10^-16^ | 48,494 |
| **NfL** |  |  |  |
| Model 1 | 2.10 (1.86 – 2.36) | < 2×10^-16^ | 48,494 |
| Model 2 | 2.05 (1.82 – 2.31) | < 2×10^-16^ | 48,494 |
| Model 3 | 1.94 (1.73 – 2.19) | < 2×10^-16^ | 48,494 |

Covariates were adjusted by model 1, 2, and 3 as described in Methods.

**Table S15.** Predictive Model Using Age or Protein Expressions and Stratified by Age Groups.

|  | **Age < 65** | | | **Age ≥ 65** | | |
| --- | --- | --- | --- | --- | --- | --- |
|  | **Age** | **GFAP + NfL** | **P** | **Age** | **GFAP + NfL** | **P** |
| **AUC** |  |  |  |  |  |  |
| All-cause dementia | 0.736 ± 0.039 | 0.758 ± 0.048 | 0.101 | 0.600 ± 0.055 | 0.665 ± 0.046 | 0.0003 |
| ADRD | 0.728 ± 0.058 | 0.786 ± 0.094 | 0.055 | 0.617 ± 0.058 | 0.700 ± 0.055 | 6.16×10^-7^ |
| VD | 0.774 ± 0.083 | 0.710 ± 0.140 | 0.082 | 0.597 ± 0.082 | 0.701 ± 0.098 | 0.002 |
| FTD | 0.677 ± 0.059 | 0.790 ± 0.045 | 0.062 | 0.550 ± 0.027 | 0.785 ± 0.088 | 0.023 |
| **C-Index** |  |  |  |  |  |  |
| All-cause dementia | 0.740 ± 0.003 | 0.760 ± 0.003 | < 2×10^-16^ | 0.592 ± 0.003 | 0.683 ± 0.003 | < 2×10^-16^ |
| ADRD | 0.731 ± 0.003 | 0.807 ± 0.004 | < 2×10^-16^ | 0.613 ± 0.003 | 0.718 ± 0.003 | < 2×10^-16^ |
| VD | 0.780 ± 0.005 | 0.746 ± 0.008 | 2.08×10^-12^ | 0.595 ± 0.005 | 0.718 ± 0.004 | < 2×10^-16^ |
| FTD | 0.682 ± 0.036 | 0.787 ± 0.020 | 0.068 | 0.552 ± 0.015 | 0.798 ± 0.057 | 0.0100 |
| **Net Reclassification Index** |  |  |  |  |  |  |
| All-cause dementia | 0.014 (0.005 – 0.027) | | - | 0.170 (0.125 – 0.216) | | - |
| ADRD | 0.012 (-0.001 – 0.029) | | - | 0.047 (0.028 – 0.072) | | - |
| VD | -0.0001 (2.35E-04 – -2.61E-05) | | - | 0.016 (-0.005 – 0.058) | | - |
| FTD | -0.0001 (-2.35E-04 – -7.83E-05) | | - | -0.0004 (-6.90E-04 – -9.85E-05) | | - |

ADRD: Alzheimer’s disease and related dementia; VD: Vascular dementia; FTD: Frontotemporal dementia.

P values were calculated by paired t-test.

**Table S16.** Predictive Model Using DRSm Combined with Protein Expressions and Stratified by Age Groups.

|  | **DRSm + GFAP + NfL** | |
| --- | --- | --- |
|  | **Age < 65** | **Age ≥ 65** |
| AUC |  |  |
| All-cause dementia | 0.834 ± 0.042 | 0.771 ± 0.045 |
| ADRD | 0.854 ± 0.074 | 0.816 ± 0.062 |
| VD | 0.872 ±0 .093 | 0.776 ± 0.092 |
| FTD | 0.758 ± 0.071 | 0.670 ± 0.058 |
| C-Index |  |  |
| All-cause dementia | 0.837 ± 0.002 | 0.783 ± 0.002 |
| ADRD | 0.870 ± 0.003 | 0.837 ± 0.002 |
| VD | 0.897 ± 0.004 | 0.812 ± 0.004 |
| FTD | 0.863 ± 0.013 | 0.866 ± 0.019 |

ADRD: Alzheimer’s disease and related dementia; VD: Vascular dementia; FTD: Frontotemporal dementia.

DRSm: Dementia Risk Score modified
